# Supplementary material for: The role of obesity and Type 2 diabetes in lung health: A systematic review (2024)
Source: PLoS One. 2026 Jan 23;21(1):e0340692. doi: 10.1371/journal.pone.0340692 (PMC12829954; doi:10.1371/journal.pone.0340692)
Supplement: S1 File — Exclusion and inclusion criteria according to the PICO method to address the research question. BMI body mass index; FEV1 forced expiratory volume in one second; FVC forced vital capacity; COPD chronic obstructive pulmonary disease; GOLD global initiative for chronic obstructive lung disease; GINA global initiative for asthma; RCT randomised controlled trial. (DOCX) [file pone.0340692.s001.docx]

**S1: PICO (Population/Problem, Intervention/Exposure, Comparison/Control, Outcome) method used to formulate the research question.**

**Research Question:** In the adult population of people with Type 2 diabetes mellitus, does hyperglycaemia and/or obesity drive COPD and asthma development?

| **Parameter** | **Inclusion Criteria** | **Exclusion Criteria** |
| --- | --- | --- |
| **Population** | Adults aged over 18 years, men or women | Under 18 years |
| **Intervention/Exposure** | Hyperglycaemia (Type 2 Diabetes) | People with any other form of diabetes (i.e., monogenic, gestational, Type 1 diabetes) |
| **Comparison/Control** | Lean or obese individuals without a diagnosis of Type 2 Diabetes or Asthma or COPD according to BMI | Obesity status measured by other means (i.e., waist circumference) |
| **Outcome** | Diagnosis of Asthma or COPD | Any other lung disease including inflammatory (i.e., Cystic Fibrosis, Idiopathic Pulmonary Fibrosis) and communicable lung diseases (i.e., Tuberculosis); Not confirmed GINA asthma or confirmed GOLD COPD; No mention of FEV1, FVC or FEV1/FVC ratio; use only of other measures of spirometry (i.e., Peak Flow) |
| **Study design** | All clinical studies (including observational, cohort studies, cross over studies etc) and RCTs. Any non-randomised studies with a corresponding control group. | Animal studies or paediatric studies. Studies not available in English. Studies focusing solely on the impact of particular drugs or COVID-19 |

Exclusion and inclusion criteria according to the PICO method to address the research question. *BMI* body mass index; *FEV1* forced expiratory volume in one second; *FVC* forced vital capacity; *COPD* chronic obstructive pulmonary disease; *GOLD* global initiative for chronic obstructive lung disease; *GINA* global initiative for asthma; *RCT* randomised controlled trial.
