# Supplementary material for: The role of obesity and Type 2 diabetes in lung health: A systematic review (2024)
Source: PLoS One. 2026 Jan 23;21(1):e0340692. doi: 10.1371/journal.pone.0340692 (PMC12829954; doi:10.1371/journal.pone.0340692)
Supplement: S2 File — Search strategies for COPD and Asthma in Ovid MEDLINE and Embase online databases with resulting article numbers before duplicates were removed. The initial COPD search (a, b) was carried out in November 2021 and the initial asthma search (c, d) in May 2022. The update checks for both asthma and COPD (e, f) were carried out in March 2023, with an additional check carried out in 2024 for articles up until January 2024 (g, h). (DOCX) [file pone.0340692.s002.docx]

**S2: Embase and Ovid MEDLINE search strategy with resulting article numbers for asthma and COPD.**

1. Embase COPD

| # | Searches | Results |
| --- | --- | --- |
| 1 | "type 2 diabet*" or "type two diabet*" or hyperglycemi* or hyperglycaemi* | 345,695 |
| 2 | non insulin dependent diabetes mellitus/ | 284,686 |
| 3 | 1 or 2 | 435,246 |
| 4 | "respiratory adj3 test*" or spirometry or COPD or "chronic obstructive pulmonary disease" or "lung function*" or "pulmonary function*" or FEV1 or FVC or "forced expiratory volume" or "forced vital capacity" | 309,474 |
| 5 | lung function test/ | 50,426 |
| 6 | chronic obstructive lung disease/ | 148,486 |
| 7 | 4 or 5 or 6 | 350,662 |
| 8 | obes* or "overweight" or over-weight or "high body mass index" or "high BMI" | 643,686 |
| 9 | obesity/ | 462,944 |
| 10 | 8 or 9 | 643,686 |
| 11 | 3 or 10 | 983,510 |
| 12 | 11 and 7 | 17,340 |
| 13 | 12 not (covid or coronavirus or sars-cov-2) | 16,617 |
| 14 | Limits: human, English, yr="2011 - 2021", article, adult <18 to 64 years> | 3,764 |

| # | Searches | Results |
| --- | --- | --- |
| 1 | "type 2 diabet*" or "type two diabet*" or hyperglycemi* or hyperglycaemi* | 214,024 |
| 2 | Diabetes Mellitus, Type 2/ | 146,972 |
| 3 | 1 or 2 | 261,967 |
| 4 | "respiratory adj3 test*" or spirometry or COPD or "chronic obstructive pulmonary disease" or "lung function*" or "pulmonary function*" or FEV1 or FVC or "forced expiratory volume" or "forced vital capacity" | 162,744 |
| 5 | Respiratory Function Tests/ | 47,938 |
| 6 | Pulmonary Disease, Chronic Obstructive/ | 44,305 |
| 7 | 4 or 5 or 6 | 190,853 |
| 8 | obes* or "overweight" or over-weight or "high body mass index" or "high BMI" | 395,933 |
| 9 | Obesity/ | 194,673 |
| 10 | 8 or 9 | 395,933 |
| 11 | 3 or 10 | 606,486 |
| 12 | 11 and 7 | 5,575 |
| 13 | 12 not (covid or coronavirus or sars-cov-2) | 5,406 |
| 14 | Limits: Humans, English, yr="2011 - 2021", "adult (19 to 44 years)" or "middle age (45 to 64 years)", journal article | 1,597 |

1. Ovid MEDLINE COPD:
2. Embase Asthma:

| # | Searches | Results |
| --- | --- | --- |
| 1 | T2D or "type 2 diabet*" or "type two diabet*" or hyperglycemi* or hyperglycaemi* | 391,292 |
| 2 | non insulin dependent diabetes mellitus/ | 327,723 |
| 3 | 1 or 2 | 492,262 |
| 4 | asthma or "lung function*" or FEV1 or FVC or FEV | 483,581 |
| 5 | lung function test/ | 54,924 |
| 6 | asthma/ | 266,170 |
| 7 | lung function/ | 95,357 |
| 8 | forced expiratory volume/ | 86,931 |
| 9 | 4 or 5 or 6 or 7 or 8 | 498,540 |
| 10 | "BMI greater than 30" or BMI>30 or obes* or "overweight" or over-weight or "high body mass index" or "high BMI" | 739,023 |
| 11 | obesity/ | 525,186 |
| 12 | 10 or 11 | 739,023 |
| 13 | 3 or 12 | 1,121,674 |
| 14 | 9 and 13 | 22,901 |
| 15 | Limits: human, English, yr="2011 - 2021", journal, adult <18 to 64 years> | 6,467 |

| # | Searches | Results |
| --- | --- | --- |
| 1 | T2D or "type 2 diabet*" or "type two diabet*" or hyperglycemi* or hyperglycaemi* | 239,414 |
| 2 | Diabetes Mellitus, Type 2/ | 167,585 |
| 3 | 1 or 2 | 290,139 |
| 4 | asthma or "lung function*" or FEV1 or FVC or FEV | 244,117 |
| 5 | Respiratory Function Tests/ | 48,947 |
| 6 | Asthma/ | 138,222 |
| 7 | Forced Expiratory Volume/ | 27,899 |
| 8 | 4 or 5 or 6 or 7 | 278,989 |
| 9 | "BMI greater than 30" or BMI>30 or obes* or "overweight" or over-weight or "high body mass index" or "high BMI" | 454,385 |
| 10 | Obesity/ | 212,796 |
| 11 | 9 or 10 | 454,385 |
| 12 | 3 or 11 | 685,945 |
| 13 | 8 and 12 | 7,984 |
| 14 | Limits: Humans, English, yr="2011 - 2021", "adult (19 to 44 years)" or "middle age (45 to 64 years)", journal article | 1,790 |

1. Ovid MEDLINE Asthma:
2. Embase Asthma and COPD 2022-2023 Update:

| # | Searches | Results | |
| --- | --- | --- | --- |
| 1 | "type 2 diabet*" or "type two diabet*" or hyperglycemi* or hyperglycaemi* | 393,982 |  |
| 2 | non insulin dependent diabetes mellitus/ | 327,723 | |
| 3 | 1 or 2 | 495,005 | |
| 4 | "respiratory adj3 test*" or spirometry or COPD or "chronic obstructive pulmonary disease" or "lung function*" or "pulmonary function*" or FEV1 or FVC or "forced expiratory volume" or "forced vital capacity" | 341,902 | |
| 5 | lung function test/ | 54,924 | |
| 6 | chronic obstructive lung disease/ | 169,471 | |
| 7 | 4 or 5 or 6 | 390,450 | |
| 8 | obes* or "overweight" or over-weight or "high body mass index" or "high BMI" | 730,866 | |
| 9 | obesity/ | 525,186 | |
| 10 | 8 or 9 | 730,866 | |
| 11 | 3 or 10 | 1,116,836 | |
| 12 | asthma | 334,329 | |
| 13 | asthma/ | 266,170 | |
| 14 | 12 or 13 | 334,329 | |
| 15 | 7 or 14 | 635,206 | |
| 16 | 11 and 15 | 32,520 | |
| 17 | 16 not (covid or coronavirus or sars-cov-2) | 30,020 | |
| 18 | Limits: human, English, yr="2022 - 2023", article, adult <18 to 64 years> | 1,224 | |

1. Ovid MEDLINE Asthma and COPD 2022-2023 Update:

| # | Searches | Results | |
| --- | --- | --- | --- |
| 1 | T2D or "type 2 diabet*" or "type two diabet*" or hyperglycemi* or hyperglycaemi* | 238,808 |  |
| 2 | Diabetes Mellitus, Type 2/ | 167,585 | |
| 3 | 1 or 2 | 289,797 | |
| 4 | "respiratory adj3 test*" or spirometry or COPD or "chronic obstructive pulmonary disease" or "lung function*" or "pulmonary function*" or FEV1 or FVC or "forced expiratory volume" or "forced vital capacity" | 176,163 | |
| 5 | Respiratory Function Tests/ | 48,947 | |
| 6 | Pulmonary Disease, Chronic Obstructive/ | 49,352 | |
| 7 | 4 or 5 or 6 | 204,655 | |
| 8 | obes* or "overweight" or over-weight or "high body mass index" or "high BMI" | 437,043 | |
| 9 | Obesity/ | 212,796 | |
| 10 | 8 or 9 | 437,043 | |
| 11 | 3 or 10 | 669,941 | |
| 12 | Asthma/ | 138,222 | |
| 13 | asthma | 195,926 | |
| 14 | 12 or 13 | 195,926 | |
| 15 | 7 or 14 | 356,536 | |
| 16 | 11 and 15 | 10,518 | |
| 17 | 16 not (covid or coronavirus or sars-cov-2) | 10,050 | |
| 18 | Limits: Humans, English, yr="2022 - 2023", "adult (19 to 44 years)" or "middle age (45 to 64 years)", journal article | 148 | |

1. Embase Asthma and COPD 2023-2024 Update:

| # | Searches | Results |
| --- | --- | --- |
| 1 | "type 2 diabet*" or "type two diabet*" or hyperglycemi* or hyperglycaemi* | 427573 |
| 2 | non insulin dependent diabetes mellitus/ | 360935 |
| 3 | 1 or 2 | 539952 |
| 4 | "respiratory adj3 test*" or spirometry or COPD or "chronic obstructive pulmonary disease" or "lung function*" or "pulmonary function*" or FEV1 or FVC or "forced expiratory volume" or "forced vital capacity" | 368516 |
| 5 | lung function test/ | 59388 |
| 6 | chronic obstructive lung disease/ | 187254 |
| 7 | 4 or 5 or 6 | 424028 |
| 8 | obes* or "over weight" or over-weight or "high body mass index" or "high BMI" | 794654 |
| 9 | obesity/ | 572043 |
| 10 | 8 or 9 | 794654 |
| 11 | 3 or 10 | 1214303 |
| 12 | asthma | 352467 |
| 13 | asthma/ | 280923 |
| 14 | 12 or 13 | 352467 |
| 15 | 7 or 14 | 680387 |
| 16 | 11 and 15 | 37478 |
| 17 | 16 not (covid or coronavirus or sars-cov-2) | 34089 |
| 18 | limit 17 to (human and english and article and adult <18 to 64 years>) | 9963 |
| 19 | limit 18 to dd=20230401-20240101 | 26 |

1. Ovid MEDLINE Asthma and COPD 2023-2024 Update:

| # | Searches | Results |
| --- | --- | --- |
| 1 | "type 2 diabet*" or "type two diabet*" or hyperglycemi* or hyperglycaemi* | 261499 |
| 2 | Diabetes Mellitus, Type 2/ | 181599 |
| 3 | 1 or 2 | 314540 |
| 4 | "respiratory adj3 test*" or spirometry or COPD or "chronic obstructive pulmonary disease" or "lung function*" or "pulmonary function*" or FEV1 or FVC or "forced expiratory volume" or "forced vital capacity" | 188493 |
| 5 | Respiratory Function Tests/ | 49463 |
| 6 | Pulmonary Disease, Chronic Obstructive/ | 53152 |
| 7 | 4 or 5 or 6 | 217181 |
| 8 | obes* or "over weight" or over-weight or "high body mass index" or "high BMI" | 473543 |
| 9 | Obesity/ | 226382 |
| 10 | 8 or 9 | 473543 |
| 11 | 3 or 10 | 726370 |
| 12 | Asthma/ | 143111 |
| 13 | asthma | 205398 |
| 14 | 12 or 13 | 205398 |
| 15 | 7 or 14 | 376187 |
| 16 | 11 and 15 | 11559 |
| 17 | 16 not (covid or coronavirus or sars-cov-2) | 10956 |
| 18 | limit 17 to (english language and humans and ("adult (19 to 44 years)" or "middle age (45 to 64 years)") and journal article) | 4125 |
| 19 | limit 18 to dt=20230401-20240101 | 88 |

Search strategies for COPD and Asthma in Ovid MEDLINE and Embase online databases with resulting article numbers before duplicates were removed. The initial COPD search (a, b) was carried out in November 2021 and the initial asthma search (c, d) in May 2022. The update checks for both asthma and COPD (e, f) were carried out in March 2023, with an additional check carried out in 2024 for articles up until January 2024 (g, h).
