# Supplementary material for: The role of obesity and Type 2 diabetes in lung health: A systematic review (2024)
Source: PLoS One. 2026 Jan 23;21(1):e0340692. doi: 10.1371/journal.pone.0340692 (PMC12829954; doi:10.1371/journal.pone.0340692)
Supplement: S3 File — Complete list of acceptable definitions of disease diagnosis for COPD, Type 2 diabetes, obesity, and asthma. Confirmation was accepted if any one of the points within each category was present, except for ‘clinical correlation’ for COPD diagnosis, ‘positive response to questionnaire asking has a medical person diagnosed you with Type 2 diabetes’ for diabetes, and ‘clinical symptoms’ for asthma diagnosis, which needed extra forms of diagnosis for confirmation. BMI body mass index; FEV1 forced expiratory volume in one second; FVC forced vital capacity; COPD chronic obstructive pulmonary disease; HbA1c glycated haemoglobin; WHO world health organisation; GOLD global initiative for chronic obstructive lung disease; GINA global initiative for asthma; MBPT methacholine bronchial provocation test; PC20 provocative concentration of methacholine causing a 20% drop in FEV1; ACQ asthma control questionnaire. (DOCX) [file pone.0340692.s003.docx]

**S3: Summary of acceptable disease definitions used in the final selected papers.**

| Term | Diagnosis Criteria |
| --- | --- |
| COPD | FEV1/FVC <70% or <50%  *AND/OR* Chronic Bronchitis, Emphysema or COPD plus two or more COPD medications  *AND/OR* Post Bronchodilator Change in FEV1 >12% or >15%  *AND/OR* FEV1 ≤80% or ≤65% of Predicted  *AND/OR*  GOLD guidelines  *AND/OR* Clinical Correlation (if paired with one or more of the above criteria) |
| Type 2 Diabetes | Plasma Glucose Concentration ≥ 200 mg/dl  *AND/OR* Fasting Plasma Glucose ≥ 126 mg/dl (7.0 mmol/L)  *AND/OR* Postprandial Blood Glucose Levels >200mg/dl  *AND/OR* HbA1c Level ≥6.5% (48 mmol/mol) or ≥7.5% (58 mmol/mol)  *AND/OR* Followed for at least 5 years for type-2 diabetes  *AND/OR* Taking Oral Hypoglycaemics  *AND/OR* Positive response to questionnaire asking has a medical person diagnosed you with type-2 diabetes (if paired with one or more of the above criteria) |
| Obesity | *WHO Western Criteria* Underweight BMI <18.5 kg/m2 Normal BMI 18.5-24.9 kg/m2 Overweight BMI 25.0-29.9 kg/m2 Obesity BMI ≥30 kg/m2  OR *WHO Asian Criteria* Underweight BMI <18.5 kg/m2 Normal BMI 18.5-22.9 kg/m2 Overweight BMI 23-24.9 kg/m2 Obesity BMI ≥25 kg/m2 |
| Asthma | MBPT of 20% decrease in FEV1 (PC20)   *AND/OR* ACQ score ≥1.5  *AND/OR* Physician Diagnosed  *AND/OR* Pre-Bronchodilator FEV1 ≥50% or ≥60% of Predicted   *AND/OR* Use of Asthma Medication  *AND/OR*  FEV1/FVC >70% of predicted  *AND/OR* GINA Guidelines   *AND/OR* Post Bronchodilator Change in FEV1 >12%  *AND/OR* Clinical Symptoms (if paired with one or more of the above criteria) |

Complete list of acceptable definitions of disease diagnosis for COPD, Type 2 diabetes, obesity, and asthma. Confirmation was accepted if any one of the points within each category was present, except for 'clinical correlation' for COPD diagnosis, 'positive response to questionnaire asking has a medical person diagnosed you with Type 2 diabetes' for diabetes, and 'clinical symptoms' for asthma diagnosis, which needed extra forms of diagnosis for confirmation.

*BMI* body mass index; *FEV1* forced expiratory volume in one second; *FVC* forced vital capacity; *COPD* chronic obstructive pulmonary disease; *HbA1c* glycated haemoglobin; *WHO* world health organisation; *GOLD* global initiative for chronic obstructive lung disease; *GINA* global initiative for asthma; *MBPT* methacholine bronchial provocation test; *PC20* provocative concentration of methacholine causing a 20% drop in FEV1; *ACQ* asthma control questionnaire.
