# Supplementary material for: The role of obesity and Type 2 diabetes in lung health: A systematic review (2024)
Source: PLoS One. 2026 Jan 23;21(1):e0340692. doi: 10.1371/journal.pone.0340692 (PMC12829954; doi:10.1371/journal.pone.0340692)
Supplement: S4 File — An adapted Newcastle-Ottawa Scale (NOS) was used for cross-sectional studies. Two stars were given to properly recorded spirometry measurements in Outcomes that followed the American Thoracic Society (ATS) and/or European Respiratory Society (ERS) standard guidelines. As the main objective of this review is to obtain lung function measurements, one star was given when spirometry values were available but the nature of how the values were obtained was not clear or if the results were not recorded properly. (DOCX) [file pone.0340692.s004.docx]

**S4: Adapted Newcastle-Ottawa Scale for cross-sectional studies.**

Note: A study can be awarded a maximum of one star for each numbered item within the Selection category. A maximum of two stars can be given for Comparability and Outcome.

**Selection:** (Maximum 3 stars)

1. Representativeness of the sample:
   1. Truly representative of the average______ in the target population. ***** (all subjects or random sampling)
   2. Somewhat representative of the average ______in the target population. ***** (non-random sampling)
   3. Selected group of users e.g., nurses, volunteers
   4. No description of the sampling strategy.
2. Selection of the control cohort:
   1. drawn from the same community as the exposed cohort (if case control) *
   2. drawn from a different source but characteristic matched (if healthy control) *
   3. no description of the derivation of the non-exposed cohort
3. Ascertainment of the exposure (risk factor)
   1. Validated measurement tool. *
   2. Non-validated measurement tool, but the tool is available or described.
   3. No description of the measurement tool.

**Comparability:** (Maximum 2 stars)

1) The subjects in different outcome groups are comparable, based on the study design or analysis. Confounding factors are controlled.

1. The study controls for ______ (the most important factor (select one)). *
2. The study control for any additional factor ______. * (These criteria could be modified to indicate specific control for a second important factor.)

**Outcome:** (Maximum 2 stars)

1. Assessment of the outcome (lung function):
2. Independent or blind assessment: properly recorded spirometry measurements **
3. Spirometry record linkage or some spirometry measurements but values not fully presented or do not indicate protocol used *
4. Spirometry not properly taken or recorded
5. No description

An adapted Newcastle-Ottawa Scale (NOS) was used for cross-sectional studies. Two stars were given to properly recorded spirometry measurements in Outcomes that followed the American Thoracic Society (ATS) and/or European Respiratory Society (ERS) standard guidelines. As the main objective of this review is to obtain lung function measurements, one star was given when spirometry values were available but the nature of how the values were obtained was not clear or if the results were not recorded properly.
