# Supplementary material for: The role of obesity and Type 2 diabetes in lung health: A systematic review (2024)
Source: PLoS One. 2026 Jan 23;21(1):e0340692. doi: 10.1371/journal.pone.0340692 (PMC12829954; doi:10.1371/journal.pone.0340692)
Supplement: S5 File — An adapted Newcastle-Ottawa Scale (NOS) was used for cohort studies. Two stars were given to properly recorded spirometry measurements in Outcomes that followed the American Thoracic Society (ATS) and/or European Respiratory Society (ERS) standard guidelines. One star was given when spirometry values were available but the nature of how the values were obtained was not clear or if the results were not recorded properly. (DOCX) [file pone.0340692.s005.docx]

**S5: Newcastle-Ottawa Scale for cohort studies.**

Note: A study can be awarded a maximum of one star for each numbered item within the Selection category. A maximum of two stars can be given for Comparability. A maximum of 4 stars can be given for Outcome category.

**Selection**

1. Representativeness of the exposed cohort
2. truly representative of the average _______________ (describe) in the community *****
3. somewhat representative of the average ______________ in the community *****
4. selected group of users eg nurses, volunteers
5. no description of the derivation of the cohort
6. Selection of the non-exposed cohort
7. drawn from the same community as the exposed cohort *****
8. drawn from a different source
9. no description of the derivation of the non-exposed cohort
10. Ascertainment of exposure
11. secure record (eg surgical records) *****
12. structured interview *****
13. written self-report
14. no description
15. Demonstration that outcome of interest was not present at start of study (diagnosis of type-2 diabetes, COPD, asthma, or obesity)
16. yes *****
17. no

**Comparability**

1. Comparability of cohorts on the basis of the design or analysis
2. study controls for _____________ (select the most important factor) *****
3. study controls for any additional factor ***** (This criteria could be modified to indicate specific control for a second important factor.)

**Outcome**

1. Assessment of outcome
2. Independent or blind assessment: properly recorded spirometry measurements ******
3. Spirometry record linkage, or some spirometry measurements but values not fully presented, or do not indicate protocol used *****
4. Spirometry not properly taken or recorded
5. No description
6. Was follow-up long enough for outcomes to occur
7. yes (select an adequate follow up period for outcome of interest) *****
8. no
9. Adequacy of follow up of cohorts
10. complete follow up - all subjects accounted for *****
11. subjects lost to follow up unlikely to introduce bias - small number lost - > ____ % (select an adequate %) follow up, or description provided of those lost) *****
12. follow up rate < ____% (select an adequate %) and no description of those lost
13. no statement

An adapted Newcastle-Ottawa Scale (NOS) was used for cohort studies. Two stars were given to properly recorded spirometry measurements in Outcomes that followed the American Thoracic Society (ATS) and/or European Respiratory Society (ERS) standard guidelines. One star was given when spirometry values were available but the nature of how the values were obtained was not clear or if the results were not recorded properly.
