# Supplementary material for: The role of obesity and Type 2 diabetes in lung health: A systematic review (2024)
Source: PLoS One. 2026 Jan 23;21(1):e0340692. doi: 10.1371/journal.pone.0340692 (PMC12829954; doi:10.1371/journal.pone.0340692)
Supplement: S6 File — (PDF) [file pone.0340692.s006.pdf]

S6: Articles assessed during the second screening.

Legend

BMI	Body Mass Index
FEV1	Forced Expiratory Volume in One Second
FVC	Forced Vital Capacity
T2DM	Type II Diabetes Mellitus
COPD	Chronic Obstructive Pulmonary Disease
SD	Standard Deviation
M/F	Male/Female
Ov	Overweight
Ob	Obese

Studies retained

Title	1st Author	Year	Study Location	Study Type	No. of Centres	Participant Numbers	Age (Mean ± SD)	BMI (Mean ± SD)	Sex (M/F *n*)	FEV (P%) (Mean ± SD)	FVC (P%) (Mean ± SD)	FEV1/FVC (L/L%) (Mean ± SD)	Smoking Status	Data Extractor
Obesity and functioning among individuals with chronic obstructive pulmonary disease (COPD)	Patricia Katz	2016	USA	Cohort	1	580 COPD Ob (BMI≥30)	58.5 ± 6.2 COPD Ob	-	224/356 COPD Ob	65 ± 22.4 COPD Ob	-	-	Mixed	RL
Study of pulmonary function tests in diabetics with COPD or asthma	Gyanshankar P. Mishra	2012	India	Cross Sectional	1	15 T2DM & COPD (Mixed BMI)<br>15 T2DM & Asthma (Mixed BMI)	61.4 ± 4.47 T2DM & COPD<br>60.27 ± 7.45 T2DM & Asthma	21.66 ± 3.95 T2DM & COPD<br>20.64 ± 2.22 T2DM & Asthma	11/4 T2DM & COPD<br>7/8 T2DM & Asthma	39.87 T2DM & COPD<br>63.07 T2DM & Asthma	65.47 T2DM & COPD<br>73.07 T2DM & Asthma	-	Unknown T2DM & COPD<br>Never T2DM & Asthma	RL
Association of chronic obstructive pulmonary disease with type 2 diabetes mellitus	Maoyun Wang	2014	China	Cross Sectional	1	37 T2DM & COPD (Mixed BMI)<br>215 T2DM Only (Mixed BMI)	-	27.1 ± 5.2 T2DM & COPD<br>25.8 ± 4.0 T2DM Only	Both	68 ± 6.1 T2DM & COPD<br>83.6 ± 6.8 T2DM Only	75.2 ± 5.1 T2DM & COPD<br>76 ± 4.6 T2DM Only	63.1 ± 4.4 T2DM & COPD<br>75.8 ± 7.3 T2DM Only	Mixed	RL
A cross-sectional study connecting obesity and pulmonary function test among young adult in Northern India region	Upendra Pandey	2021	India	Cross Sectional	1	43 Lean (BMI 18.5-22.9)	25.96 ± 6.09 Lean	21.89 ± 1.98 Lean	43/0 Lean	-	-	70.80 ± 2.44 Lean	Never	RL
A study on pulmonary function parameters in type 2 diabetes mellitus.	R Elizabeth Rani	2019	India	Cross Sectional	1	20 T2DM Only (Mixed BMI)	50.5 ± 8.25 T2DM Only	25.86 ± 3.1 T2DM Only	Both	67.5 ± 10.88 T2DM Only	58.9 ± 8.8 T2DM Only	-	Never	RL
Alteration of pulmonary function in diabetic nephropathy	Gita Shafiee	2013	Iran	Cross Sectional	1	80 T2DM Only (Mixed BMI)	53.6 ± 11.9 T2DM Only	28.8 ± 4.1 T2DM Only	55/25 T2DM Only	107.07 ± 15.98 T2DM Only	107.59 ± 16.53 T2DM Only	-	Never	RL
Alveolar Gas Exchange and Pulmonary Functions in Patients with Type II Diabetes Mellitus	S Anandhalakshmi	2013	India	Cross Sectional	1	30 T2DM Only (Mixed BMI)	44.8 ± 8.9 T2DM Only	26.06 ± 4.03 T2DM Only	Both	-	-	96.1 ± 10.1 T2DM Only	Never	RL
Assessment of Pulmonary Functions in Obese Young Adults	Swapnil Bhirange	2020	India	Cross Sectional	2	40 Lean (BMI 18.5-23)<br>80 Ov (BMI 25-29.9)	20.6 ± 1.4 Lean<br>20.8 ± 1.5 Ov	20.7 ± 1.8 Lean<br>26.8 ± 1.3 Ov	20/20 Lean<br>40/40 Ov	-	-	95.75 ± 4.08 Lean<br>87.84 ± 5.82 Ov	Mixed	RL
Assessment of pulmonary functions in type 2 diabetes mellitus: Its correlation with glycemic control and body mass index	Santosh V. Chidri	2020	India	Cross Sectional	1	100 T2DM Only (Mixed BMI)	43.54 ± 6.21 T2DM Only	26.58 ± 2.89 T2DM Only	Both	78.53 ± 10.26 T2DM Only	81.22 ± 10.08 T2DM Only	84.06 ± 4.72 T2DM Only	Never	RL
Association of body mass index with pulmonary function in overweight young adults	Nida Nowreen	2019	India	Cross Sectional	1	100 Ov (BMI 25-29.9)	19.6 ± 1.5 Ov	26.89 ± 2.6 Ov	50/50 Ov	-	-	86.95 ± 7.18 Ov	Never	RL
Body Mass Index and Dynamic Lung Volumes in Office Workers	Sohail Attaur-Rasool	2012	Pakistan	Cross Sectional	1	78 Lean (BMI 18.5-24.9)<br>102 Ov (BMI 25-29.9)<br>45 Ob (BMI≥30)	38.81 ± 7.72 Lean<br>41.30 ± 8.0 Ov<br>42.40 ± 7.27 Ob	22.25 ±1.92 Lean<br>27.24 ± 1.49 Ov<br>32.42 ± 2.55 Ob	Both	96.85 ± 11.11 Lean<br>92.86 ± 12.74 Ov<br>89.39 ± 12.65 Ob	97.97 ± 10.92 Lean<br>93.89 ± 12.68 Ov<br>89.57 ± 10.96 Ob	82.86 ± 4.83 Lean<br>81.99 ± 5.90 Ov<br>82.52 ± 6.19 Ob	Never	RL
Comparative study on differences in lung parameter between the obese and non obese collegiate sedentary students	Jessie Kho S'Sia Yen	2018	Malaysia	Cross Sectional	1	40 Lean (BMI 18.5-25.9)<br>40 Ob (BMI>30)	-	-	Both	104 ± 7.77 Lean<br>86.9 ± 4.09 Ob	-	118 ± 5.12 Lean<br>100 ± 4.76 Ob	Never	RL
Duration of type 2 diabetes mellitus and pulmonary function tests: a correlative study	Swati Mittal	2020	India	Cross Sectional	1	101 T2DM Only (Mixed BMI)	51.5 ± 8.4 T2DM Only	26.6 ± 3.1 T2DM Only	52/49 T2DM Only	62.74 T2DM	62.85 T2DM	-	Never	RL
Effect of Body Fat Distribution on Pulmonary Functions in Young Healthy Obese Students	Sowmya Timmanna Koraddi	2015	India	Cross Sectional	1	50 Lean (BMI 18.5-24.9)<br>50 Ob (BMI>30)	20.3 ± 2.1 Lean<br>20.7 ± 2.3 Obesity Only	21.1 ± 1.4 Lean<br>30.4 ± 1.1 Ob	32/18 Lean<br>23/27 Ob	92.07 ± 7.09 Lean<br>88.5 ± 12.96 Ob	-	-	Never	SG
Effect of Body Mass Index on respiratory parameters:<br>A cross-sectional analytical Study	Urooj Bhatti	2019	Pakistan	Cross Sectional	1	47 Lean (BMI 18.5-24.9)<br>55 Ov (BMI 25-29.9)<br>44 Ob (BMI≥30)	-	-	Both	-	-	87.94 ± 7.83 Lean<br>86.4 ± 10.4 Ov<br>87.1 ± 8.25 Ob	Never	RL
Effect of duration of diabetes on pulmonary functions in non-smoker type-2 diabetes mellitus	Tanya Saxena	2020	India	Cross Sectional	1	70 T2DM Only (Mixed BMI)	50.9 ± 6.95 T2DM Only	-	37/33 T2DM Only	82.03 ± 11.38 T2DM Only	-	-	Never	RL
Effect of Glucose Improvement on Spirometric Maneuvers in Patients With Type 2 Diabetes: The Sweet Breath Study	Liliana Gutierrez-Carrasquilla	2019	Spain	Prospective Interventional	1	60 T2DM Only (Mixed BMI)	58.1 ± 6.4 T2DM Only	32.4 ± 6.1 T2DM Only	47/13 T2DM Only	76.5 ± 13.8 T2DM Only	77.8 ± 11.3 T2DM Only	85.3 ± 14.0 T2DM Only	Mixed	RL
Effect of Glycated Hemoglobin (HbA1c) and Duration of Disease on Lung Functions in Type 2 Diabetic Patients	Hawra Bin Maan	2021	Saudi Arabia	Case- Controlled Cross Sectional	1	101 T2DM Only (BMI<30)	55.50 ± 5.99 T2DM Only	25.04 ± 2.11 T2DM Only	71/30 T2DM Only	-	-	88.47 ± 6.98 T2DM Only	Never	RL
Effect of glycemic status on lung function tests in type 2 diabetes mellitus	S N Naithok Jamatia	2014	India	Cross Sectional	1	30 T2DM Only (Mixed BMI)	57.66 ± 4.68 T2DM Only	23.52 ± 3.61 T2DM Only	19/11 T2DM Only	-	-	52 ± 0.46 T2DM Only	Never	RL
Effect of Obesity and Hypertension on Pulmonary Functions	Suresh Nayak B.	2014	India	Cross Sectional	1	40 Ob (BMI≥30)	49.49 ± 6.9 Ob	33.04 ± 2.6 Ob	40/0 Ob	101.79 ± 17.08 Ob	-	-	Never	RL
Effect of weight reduction on obese patients with COPD and bronchial asthma	Basem I. El-Shafey	2015	Egypt	Cross Sectional	1	30 COPD Ob (BMI>30)<br>30 Asthma Ob (BMI>30)	47.2 ± 1.3 COPD Ob<br>45.6 ± 2.6 Asthma Ob	34.6 ± 2.19 COPD Ob<br>36.6 ± 2.97 Asthma Ob	17/13 COPD Ob<br>15/15 Asthma Ob	47.6 ± 3.21 COPD Ob<br>69 ± 5.74 Asthma Ob	77.2 ± 6.53 COPD Ob<br>78 ± 4.64 Asthma Ob	48.2 ± 3.11 COPD Ob<br>89 ± 4.00 Asthma Ob	Never	SG
Effects of progressive increase in body weight on lung function in six groups of body mass index	Saulo Maia D'Ávila Melo	2011	Brazil	Cross Sectional	1	114 Ob (BMI≥30)	36.97 ± 11.25 Ob	-	42/72 Ob	86.87 ± 11.16 Ob	87.02 ± 11.34 Ob	81.81 ± 5.24 Ob	Mixed	SG
Impact Of Obesity on Pulmonary Functions Among Young Non-Smoker Healthy Female of Shah Alam, Malaysia	Aniruddha Bhattacharjee	2018	Malaysia	Cross Sectional	1	50 Lean (BMI 18.5-24.99)<br>50 Ob (BMI>30)	21.24 ± 2.18 Lean<br>20.68 ± 2.08 Ob	22.81 ± 2.27 Lean<br>32.31 ± 5.23 Ob	0/50 Lean<br>0/50 Ob	-	-	95.29 ± 7.32 Lean<br>89.04 ± 12.71 Ob	Never	RL
Impact of overweight and obesity on ventilatory function among male medical students	Rajab Ali Khawaja	2011	Saudi Arabia	Cross Sectional	1	68 Lean (BMI 18.5-24.9)<br>19 Ov (BMI 25-29.9)<br>26 Ob (BMI>30)	19.78 ± 0.75 Lean<br>19.63 ± 0.60 Ov<br>20.00 ± 0.69 Ob	-	68/0 Lean<br>19/0 Ov<br>26/0 Ob	-	-	90.09 ± 4.96 Lean<br>82.90 ± 6.38 Ov<br>85.94 ± 4.97 Ob	Never	RL
Lung Functions in Type 2 Diabetes Mellitus	Anjali N. Shete	2014	India	Cross Sectional Observational	1	30 T2DM Only (Mixed BMI)	48.3 ± 7.97 T2DM Only	27.68 ± 4.12 T2DM Only	Both	-	-	88.77 ± 1.67 T2DM Only	Never	RL
Metabolic Determinants of Impaired Pulmonary Function in Patients with Newly Diagnosed Type 2 Diabetes Mellitus	Martin Röhling	2018	Germany	Prospective Observational	1	34 T2DM Only (Mixed BMI)	53 ± 9 T2DM Only	30.8 ± 5.6 T2DM Only	21/13 T2DM Only	77.3 ± 15.6 T2DM Only	96.8 ± 22.0 T2DM Only	-	Mixed	RL
Non-linear association of anthropometric measurements and pulmonary function	Rui-Heng Zhang	2021	China<br>(USA Data)	Cross Sectional	Multiple	2198 Lean (BMI 18.5-24.9)<br>2557 Ov (BMI 25-29.9)<br>2483 Ob (BMI≥30)	40.4 Lean<br>44.9 Ov<br>44.0 Ob	22.42 Lean<br>27.36 Ov<br>35.26 Ob	949/1249 Lean<br>1470/1087 Ov<br>1216/2483 Ob	98.0 Lean<br>98.6 Ov<br>96.5 Ob	101.7 Lean<br>101.5 Ov<br>98.3 Ob	79.5 Lean<br>77.8 Ov<br>79.0 Ob	Mixed	SG
Obesity and Pulmonary Functions in Young Non Smoker Male of Shah Alam, Malaysia	Aniruddha Bhattacharjee	2018	Malaysia	Cross Sectional	1	50 Lean (BMI 18.5-24.9)<br>50 Ob (BMI>30)	21.16 ± 2.17 Lean<br>21.2 ± 0.05 Ob	22.32 ± 2.35 Lean<br>31.10 ± 3.74 Ob	50/0 Lean<br>50/0 Ob	-	-	91.58 ± 8.36 Lean<br>78.62 ± 18.83 Ob	Never	RL
Pulmonary function changes in diabetic lung	I. Amal Abd El-Azeem	2013	Egypt	Cross Sectional	14	30 T2DM Only (BMI<30)	-	-	Both	-	-	85.1 ± 0.1 T2DM Only	Never	RL
Pulmonary Function Tests and Their Associated Factors Among Type 2 Diabetic Patients at Jimma Medical Center, in 2019; Comparative Cross Sectional Study	Dereje Gemeda Tesema	2020	Ethiopia	Cross Sectional	1	145 T2DM Only (Mixed BMI)	52.2 ± 9.75 T2DM Only	26.5 ± 3.12 T2DM Only	80/65 T2DM Only	76.4 ± 13.4 T2DM Only	73.7 ± 13.8 T2DM Only	78.9 ± 11.4 T2DM Only	Never	RL
Pulmonary function tests in type 2 diabetes mellitus and their association with glycemic control and duration of the disease	Swati H. Shah	2013	India	Cross Sectional	1	60 T2DM Only (Mixed BMI)	53.90 ± 8.45 T2DM Only	-	60/0 T2DM Only	78.98 ± 14.09 T2DM Only	77.97 ± 12.99 T2DM Only	-	Never	RL
Pulmonary function tests in type 2 diabetes mellitus and their association with glycemic status and insulin resistance	Sarita Bajaj	2020	India	Case-Control	1	100 T2DM Only (Mixed BMI)	-	-	Both	78.71 ± 1.51 T2DM Only	67.48 ± 14.06 T2DM Only	121.7 T2DM Only	Never	RL

Title	1st Author	Year	Study Location	Study Type	No. of Centres	Participant Numbers	Age (Mean ± SD)	BMI (Mean ± SD)	Sex (M/F *n*)	FEV (P%) (Mean ± SD)	FVC (P%) (Mean ± SD)	FEV1/FVC (L/L%) (Mean ± SD)	Smoking Status	Data Extractor
Pulmonary Function Tests in Type 2 Diabetics and Non-Diabetic People -A Comparative Study	Aparna A.	2013	India	Cross Sectional	1	40 T2DM Only (Mixed BMI)	49.13 ± 1.97 T2DM Only	25.17 ± 1.64 T2DM Only	22/18 T2DM Only	-	-	83.25 ± 3.26 T2DM Only	Never	RL
Reduced lung function is independently associated with increased risk of type 2 diabetes in Korean men	Chang-Hee Kwon	2012	Republic of Korea	Longitudinal Cohort	1	207 T2DM Only (Mixed BMI)	42.6 ± 5.6 T2DM Only	26.7 ± 3.3 T2DM Only	207/0 T2DM Only	103.5 ± 19.1 T2DM Only	95.2 ± 15.3 T2DM Only	-	Mixed	RL
Reduction In Lung Functions in Type-2 Diabetes in Indian Population: Correlation With Glycemic Status	Anand R. Dharwadkar	2011	India	Cross Sectional	1	40 T2DM Only (Mixed BMI)	52.3± 7.6 T2DM Only	22.69± 3.41 T2DM Only	25/15 T2DM Only	-	-	67.44 ± 16.51 T2DM Only	Never	RL
Respiratory function in type II diabetes mellitus	Mokhles Abdel Fadil Zineldin	2015	Egypt	Cross Sectional	1	45 T2DM Only (Mixed BMI)	51.11 ± 6.16 T2DM Only	24.49 ± 0.81 T2DM Only	45/0 T2DM Only	73.42 ± 3.77 T2DM Only	75.04 ± 3.81 T2DM Only	97.84 ± 1.74 T2DM Only	Never	SG
Restrictive pulmonary deficit is associated with inflammation in suboptimally controlled obese diabetics	Lexley M. Pinto Pereira	2013	West Indies	Cross Sectional	2	109 T2DM Only (Mixed BMI)	55.62 ± 11.27 T2DM Only	29.3 ± 6.65 T2DM Only	47/62 T2DM Only	88.43 ± 15.44 T2DM Only	82.24 ± 13.76 T2DM Only	85.13 ± 5.12 T2DM Only	Mixed	RL
Study of lung function in patients of type 2 diabetes mellitus	Aparajita Maji Mandal	2021	India	Cross Sectional	1	100 T2DM Only (BMI≤35)	46.1 ± 8.42 T2DM Only	25.59 ± 2.85 T2DM Only	72/28 T2DM Only	84.91 ± 3.95 T2DM Only	-	-	Never	RL
The Effect of Obesity on Pulmonary Function Testing Among the Jordanian Population	Walid Al-Qerem	2018	Jordan	Cross Sectional	1	183 Lean (BMI 18.5-24.9)<br>202 Ob (BMI>30)	30.08 ± 11.67 Lean<br>31.30 ± 12.67 Ob	22 ± 1.9 Lean<br>33.79 ± 5.08 Ob	106/77 Lean<br>142/60 Ob	-	-	91.44 ± 6.93 Lean<br>90.41 ± 9.33 Ob	Never	RL
The effects of body mass index on spirometry tests among adults in Xi'an, China	Shengyu Wang	2017	China	Cross Sectional	1	457 Lean (BMI<24)	-	-	Both	-	-	75 ± 1 Lean	Mixed	SG
The Relationship Between Anthropometric Measures, Blood Gases, and Lung Function in Morbidly Obese White Subjects	Anne-Marie Gabrielsen	2011	Norway	Cross Sectional	1	149 Ob (BMI≥35)	43 ± 11 Ob	45.0 ± 6.3 Ob	35/114 Ob	97 ± 15 Ob	103 ± 15 Ob	-	Mixed	RL
The study of pulmonary functions tests and fat distribution in overweight and obese adult males	Divyesh S. Vadasmiya	2019	India	Cross Sectional	1	30 Lean (BMI<25)	30.26 ± 7.95 Lean	22.64 ± 1.79 Lean	30/0 Lean	82.24 ± 3.66 Lean	96.32 ± 2.86 Lean	85.36 ± 4.86 Lean	Never	RL
Type II diabetes mellitus is associated with decreased measures of lung function in a clinical setting	Oana L. Klein	2011	USA	Cross Sectional Retrospective	1	76 T2DM Only (Mixed BMI)	63 T2DM Only	34.2 T2DM Only	33/43 T2DM Only	75.3 T2DM Only	71.2 T2DM Only	-	Mixed	SG
A prospective study on physical performance of Chinese chronic obstructive pulmonary disease males with type 2 diabetes	Junhong Liu	2021	China	Prospective Cohort	1	55 COPD & T2DM (Mixed BMI)	70.5±6.7 COPD & T2DM	26.2±3.0 COPD & T2DM	55/0 COPD & T2DM	49.8 ± 6.9 COPD & T2DM	-	59.8 ± 5.3 COPD & T2DM	Mixed	RL
Determinants of exercise capacity in obese and non-obese COPD patients	Diego A. Rodrıguez	2014	Spain	Cross Sectional	9	108 COPD Ob (BMI≥30)	68 ± 8 COPD Ob	33.2 ± 2.8 COPD Ob	102/6 COPD Ob	58 ± 8 COPD Ob	-	-	Mixed	RL
Diabetes Mellitus Type 2 in Hospitalized COPD Patients: Impact on Quality of Life and Lung Function	Evgeni V. Mekov	2016	Bulgaria	Cross Sectional	1	53 COPD & T2DM (Mixed BMI)	-	-	40/13 COPD & T2DM	55.02 COPD & T2DM	72.62 COPD & T2DM	57 COPD & T2DM	Mixed	RL
Effect of obesity on respiratory mechanics during rest and exercise in COPD	Josuel Ora	2011	Canada	Cross Sectional	1	12 COPD Lean (BMI 18.5–24.9)<br>12 COPD Ob (BMI 30.0–34.9)	68 ± 8 COPD Lean<br>68 ± 4 COPD Ob	23.4 ± 1.8 COPD Lean<br>32.2 ± 1.2 COPD Ob	6/6 COPD Lean<br>6/6 COPD Ob	59 ± 17 COPD Lean<br>60 ± 13 COPD Ob	95 ± 16 COPD Lean<br>92 ± 20 COPD Ob	42 ± 8 COPD Lean<br>47 ± 12 COPD Ob	Mixed	RL
Grading the severity of obstruction in patients with Chronic Obstructive Pulmonary Disease and morbid obesity	Nicola Barbarito	2013	Italy	Cross Sectional	1	16 COPD Lean (BMI 22-24.9)<br>17 COPD Ob (BMI≥40)	73 ± 8 COPD Lean<br>69 ± 7 COPD Ob	23 ± 1 COPD Lean<br>47 ± 6 COPD Ob	10/6 COPD Lean<br>7/10 COPD Ob	49 ± 18 COPD Lean<br>57 ± 18 COPD Ob	82 ± 21 COPD Lean<br>73 ± 19 COPD Ob	50 ± 9 COPD Lean<br>62 ± 7 COPD Ob	Current/Previous	RL
Impact of diabetes mellitus on the risk of severe exacerbation in patients with chronic obstructive pulmonary disease	Juan M. Figueira-Gonçalves	2020	Spain	Prospective Observational Cohort	1	47 COPD & T2DM (Mixed BMI)	73.79 ± 8.76 COPD & T2DM	30.34 ± 4.2 COPD & T2DM	44/3 COPD & T2DM	47.98 ± 11.14 COPD & T2DM	73.98 ± 16.27 COPD & T2DM	51.62 ± 12.16 COPD & T2DM	Current/Previous	RL
Obesity in chronic obstructive pulmonary disease: Is fatter really better?	Roxana G. Galesanu	2014	Canada	Cohort	1	91 COPD Lean (BMI<25)	65 ± 9 COPD Lean	22 ± 3 COPD Lean	76/15 COPD Lean	35 ± 12 COPD Lean	70 ± 17 COPD Lean	40 ± 9 COPD Lean	Current/Previous	RL
The impact of sex and BMI on the clinical course of COPD and bronchial asthma	Krzysztof Wytrychowski	2016	Poland	Cross Sectional	1	29 COPD Ob (BMI>30)<br>28 Asthma Ob (BMI>30)	68.3 ± 7.8 COPD Ob<br>52.3 ± 12.8 Asthma Ob	33.4 ± 3.1 COPD Ob<br>33.6 ± 3.4 Asthma Ob	16/13 COPD Ob<br>10/18 Asthma Ob	52.9 ± 14.9 COPD Ob<br>66.1 ± 16.4 Asthma Ob	-	-	Mixed	RL
Correlation of Pulmonary Function Tests with Anthropometry and Glycaemic Control in Type 2 Diabetes Mellitus: A Cross-sectional Study	Aiswarya Roy Karintholil	2021	India	Cross Sectional	1	80 T2DM Only (Mixed BMI)	58.43 ± 10.73 T2DM Only	24.71 ± 3.52 T2DM Only	45/35 T2DM Only	62.01 ± 12.79 T2DM Only	60.29 ± 11.39 T2DM Only	80 ± 8 T2DM Only	Never	RL
Decline of the lung function and quality of glycemic control in type 2 diabetes mellitus	Leonello Fuso	2015	Italy	Longitudinal Cohort	1	45 T2DM Only (Mixed BMI)	63.81 ± 6.36 T2DM Only	29.46 ± 4.99 T2DM Only	28/17 T2DM Only	97.43 ± 18.29 T2DM Only	101.82 ± 16.96 T2DM Only	76.04 ± 6.11 T2DM Only	Never	RL
Effect of Type 2 Diabetes Mellitus on Pulmonary Function	H. Huang	2014	China	Cross Sectional Retrospective	1	292 T2DM Only (Mixed BMI)	66.85 ± 9.56 T2DM Only	23.94 ± 3.81 T2DM Only	181/111 T2DM Only	89.14 ± 17.65 T2DM Only	85.02 ± 12.65 T2DM Only	82.92 ± 9.13 T2DM Only	Never	CK
Gender Differences and Obesity Influence on Pulmonary Function Parameters	Rahimah Zakaria	2019	Malaysia	Cross Sectional Retrospective	1	53 Lean (BMI 18.5-24.9)<br>43 Ov (BMI 25-29.9)<br>17 Ob (BMI≥30)	61.0 ± 14.2 Lean<br>56.1 ± 12.3 Ov<br>56.8 ± 11.2 Ob	-	36/17 Lean<br>30/13 Ov<br>9/8 Ob	63.4 ± 24.4 Lean<br>67.4 ± 20.4 Ov<br>62.9 ± 18.4 Ob	66.7 ± 22.3 Lean<br>69.8 ± 18.7 Ov<br>60.5 ± 16.6 Ob	76.0 ± 14.4 Lean<br>78.2 ± 10.9 Ov<br>84.6 ± 5.9 Ob	Mixed	RL
Mortality and Exacerbation Risk by Body Mass Index in Patients with COPD in TIOSPIR and UPLIFT	Nirupama Putcha	2022	USA<br>(TIOSPIR 9 countries)	*Post hoc* analysis (Cross Sectional)	117 TIOSPIR<br>490 UPLIFT	5721 COPD Lean (BMI 20-24.9)<br>5509 COPD Ov (BMI 25-29.9)<br>3823 COPD Ob (BMI≥30)	65.5 ± 9.2 COPD Lean<br>65.4 ± 9.0 COPD Ov<br>63.94 ± 8.71 COPD Ob	-	4169/1552 COPD Lean<br>4037/1472 COPD Ov<br>2582/1241 COPD Ob	47.3 ± 14.5 COPD Lean<br>51.0 ± 13.9 COPD Ov<br>52.52 ± 13.61 COPD Ob	79.5 ± 20.4 COPD Lean<br>80.1 ± 19.2 COPD Ov<br>77.73 ± 18.31 COPD Ob	50 ± 10 COPD Lean<br>50 ± 10 COPD Ov<br>53.14 ± 11.02 COPD Ob	Mixed	RL
Airway hyperresponsiveness is negatively associated with obesity or overweight status in patients with asthma	Jae-Woo Kwon	2012	Republic of Korea	Cohort	11	546 Asthma Lean (BMI 18.5-24.9)<br>233 Asthma Ov (BMI 25-29.9)<br>42 Asthma Ob (BMI≥30)	45.5 ± 15.5 Asthma Lean<br>49.5 ± 15.9 Asthma Ov<br>53.4 ± 14.3 Asthma Ob	-	238/308 Asthma Lean<br>126/107 Asthma Ov<br>11/31 Asthma Ob	85.3 ± 19.0 Asthma Lean<br>87.0 ± 17.7 Asthma Ov<br>81.5 ± 19.1 Asthma Ob	91.9 ± 16.6 Asthma Lean<br>91.2 ± 16.1 Asthma Ov<br>84.6 ± 15.9 Asthma Ob	79.8 ± 5.9 Asthma Lean<br>78.1 ± 5.9 Asthma Ov<br>79.2 ± 4.7 Asthma Ob	Mixed	RL
Asthma diagnosis is not associated with obesity in a population of adults from Madrid	P Barranco	2011	Spain	Cross Sectional	1	151 Asthma Lean (BMI<25)<br>72 Asthma Ov (BMI 25-29.9)<br>28 Asthma Ob (BMI≥30)	-	-	34/117 Asthma Lean<br>37/35 Asthma Ov<br>12/16 Asthma Ob	83.7 ± 11 Asthma Lean<br>81.01 ± 9.6 Asthma Ov<br>83 ± 14.3 Asthma Ob	-	83 Asthma Lean<br>81 Asthma Ov<br>83 Asthma Ob	Mixed	SG
Baseline of visceral fat area and decreased body weight correlate with improved pulmonary function after Roux-en-Y gastric bypass in Chinese obese patients with BMI 28-35 kg/m2 and type 2 diabetes: a 6-month follow-up	Yinfang Tu	2015	China	Cross Sectional Retrospective	1	32 T2DM Only (Mixed BMI)	45.09 ± 11.26 T2DM Only	30.7 ± 3.5 T2DM Only	14/18 T2DM Only	91.6 T2DM Only	87.7 T2DM Only	86.4 T2DM Only	Mixed	SG
Effect of obesity on asthma phenotype is dependent upon asthma severity	Stacy Raviv	2011	USA	Cross Sectional	38	56 Asthma Lean (BMI 20-24.9)<br>69 Asthma Ov (BMI 25-29.9)<br>101 Asthma Ob (BMI≥30)	35.3 ± 13.6 Asthma Lean<br>37.0 ± 13.6 Asthma Ov<br>38.6 ± 10.9 Asthma Ob	22.6 ± 1.4 Asthma Lean<br>27.3 ± 1.6 Asthma Ov<br>37.3 ± 6.4 Asthma Ob	18/38 Asthma Lean<br>26/43 Asthma Ov<br>23/78 Asthma Ob	91.9 ± 13.4 Asthma Lean<br>90.0 ± 11.4 Asthma Ov<br>88.0 ± 10.7 Asthma Ob	100.6 ± 13.7 Asthma Lean<br>98.6 ± 12.4 Asthma Ov<br>93.8 ± 12.5 Asthma Ob	76 ± 7 Asthma Lean<br>75 ± 7 Asthma Ov<br>78 ± 7 Asthma Ob	Never	SG
Functional lung rejuvenation in obese patients after bariatric surgery	Saulo Maia Davila Melo	2016	Brazil	Prospective Longitudinal Cohort	1	43 Ob (BMI≥35)	38.72 ± 10.12 Ob	44.18 ± 7.45 Ob	13/30 Ob	82.41 ± 8.13 Ob	80.53 ± 8.33 Ob	82.90 ± 7.69 Ob	Never	SG
Influence of body mass indexes on response to treatment in acute asthma	Ebrahim Razi	2014	Iran	Cross Sectional	1	107 Asthma Lean (BMI<25)	39.50 ± 14.25 Asthma Lean	22.05 ± 2.19 Asthma Lean	71/36 Asthma Lean	49 ± 13.67 Asthma Lean	65.74 ± 14.43 Asthma Lean	62.45 ± 9.61 Asthma Lean	-	RL
Lung age in women with morbid obesity	Fabiana Sobral Peixoto-Souza	2013	Brazil	Cross Sectional	1	37 Lean (BMI 18.5-24.9)<br>72 Ob (BMI 40-55)	34.9 ± 7.6 Lean<br>34.6 ± 6.8 Ob	22.7 ± 1.9 Lean<br>45.8 ± 5.4 Ob	0/37 Lean<br>0/72 Ob	106.8 ± 11.4 Lean<br>96.3 ± 12.3 Ob	103.1 ± 9.6 Lean<br>96.0 ± 13.1 Ob	-	Mixed	RL
Observational study of the effect of obesity on lung volumes	Joerg Steier	2014	UK	Cross Sectional	1	9 Lean (BMI 18.5-24.9)<br>9 Ob(BMI>30)	38 ± 11 Lean<br>45 ± 13 Ob	23.2 ± 1.6 Lean<br>46.8 ± 17.2 Ob	5/4 Lean<br>4/5 Ob	119.6 ± 17.8 Lean<br>97.4 ± 24.7 Ob	125.2 ± 17.3 Lean<br>96.8 ± 19.7 Ob	82.5 ± 4.2 Lean<br>79.6 ± 7.3 Ob	Mixed	RL

Title	1st Author	Year	Study Location	Study Type	No. of Centres	Participant Numbers	Age (Mean ± SD)	BMI (Mean ± SD)	Sex (M/F *n*)	FEV (P%) (Mean ± SD)	FVC (P%) (Mean ± SD)	FEV1/FVC (L/L%) (Mean ± SD)	Smoking Status	Data Extractor
Overweight is Associated with Airflow Obstruction and Poor Disease Control but Not with Exhaled Nitric Oxide Change in an Asthmatic Population	Roberta Pisi	2012	Italy	Cohort	1	203 Asthma Lean (BMI 18.5-24.9)<br>145 Asthma Ov (BMI 25-30)	37 ± 15 Asthma Lean<br>48 ± 16 Asthma Ov	-	Both	97 ± 15 Asthma Lean<br>91 ± 19 Asthma Ov	107 ± 15 Asthma Lean<br>103 ± 18 Asthma Ov	77 ± 9 Asthma Lean<br>73 ± 9 Asthma Ov	Mixed	RL
Reduced pulmonary functions and respiratory muscle strength in Type 2 diabetes mellitus and its association with glycemic control	T. Al-Khlaiwi	2021	Saudi Arabia	Cross Sectional	2	110 T2DM Only (Mixed BMI)	45.51 ± 13.03 T2DM Only	28.55 ± 4.89 T2DM Only	71/39 T2DM Only	84.91 ± 9.1 T2DM Only	-	-	Never	CK
Relationship between pulmonary function and albuminuria in type 2 diabetic patients with preserved renal function	Yun-Yun He	2020	China	Cross Sectional	1	326 T2DM Only (Mixed BMI)	53.52 ± 11.57 T2DM Only	27.07 ± 3.86 T2DM Only	222/104 T2DM Only	98.30 ± 14.41 T2DM Only	102.78 ± 14.22 T2DM Only	78.37 ± 5.46 T2DM Only	Mixed	RL
Serum Surfactant Protein D as a Biomarker for Measuring Lung Involvement in Obese Patients With Type 2 Diabetes	Carolina Lo´ pez-Cano	2017	Spain	Case-Control	1	98 Ob (BMI≥30)<br>49 T2DM Ob (BMI≥30)	48.5 ± 9.4 Ob<br>51.3 ± 10.6 T2DM Ob	42.6 ± 6.7 Ob<br>42.0 ± 7.7 T2DM Ob	24/74 Ob<br>12/37 T2DM Ob	101.1 ± 13.1 Ob<br>82.9 ± 25.9 T2DM Ob	92.9 ± 11.0 Ob<br>79.7 ± 21.7 T2DM Ob	-	Never	SG
Spirometric values in elderly asthmatic patients are not influenced by obesity	R. C. Agondi	2012	Brazil	Cross Sectional	1	153 Asthma Lean (BMI 18.5-24.9)<br>153 Asthma Ov (BMI 25-29.9)<br>145 Asthma Ob (BMI≥30)	45.8 ± 17.8 Asthma Lean<br>50.4 ± 14.6 Asthma Ov<br>51.6 ± 13.6 Asthma Ob	22.7 ± 1.71 Asthma Lean<br>27.3 ± 1.33 Asthma Ov<br>34.0 ± 3.73 Asthma Ob	45/108 Asthma Lean<br>35/118 Asthma Ov<br>22/123 Asthma Ob	81.2 ± 19.3 Asthma Lean<br>79.9 ± 17.1 Asthma Ov<br>74.9 ± 17.7 Asthma Ob	93.0 ± 16.7 Asthma Lean<br>91.2 ± 15.1 Asthma Ov<br>86.8 ± 15.0 Asthma Ob	72 ± 10 Asthma Lean<br>71 ± 10 Asthma Ov<br>70 ± 10 Asthma Ob	-	SG
Total and Compartmental Chest Wall Volumes, Lung Function, and Respiratory Muscle Strength in Individuals with Abdominal Obesity: Effects of Body Positions	Rattanaporn Sonpeayung	2019	Thailand	Cross Sectional	1	20 Lean (BMI 18.5-22.9)	27.2 ± 3.90 Lean	21.62 ± 0.95 Lean	20/0 Lean	-	-	82.78 Lean	-	CK
Association body mass index and spirometric lung function in chronic obstructive pulmonary disease (COPD) patients attending RIMS Hospital, Manipur	Awungshi Jannie Shimray	2014	India	Cross Sectional	2	27 COPD Lean (BMI≥18.5)	-	-	Both	70.91 ± 24.9 COPD Lean	71.3 ± 10.46 COPD Lean	-	Current/Previous	RL
Surgically induced weight loss, including reduction in waist circumference, is associated with improved pulmonary function in obese patients	Yu-Feng Wei	2011	Taiwan	Cohort	1	94 Ob (BMI>32)	31.2 ± 9.8 Ob	43.4 ± 7.3 Ob	Both	91.8 ± 15.3 Ob	92.8 ± 15 Ob	84.1 ± 4.5 Ob	-	SG
Obesity is a determinant of asthma control independent of inflammation and lung mechanics	Claude S. Farah	2011	Australia	Cohort	1	20 Asthma Lean (BMI 18.5-24.9)<br>14 Asthma Ov (BMI 25-29.9)<br>15 Asthma Ob (BMI≥30)	29 Asthma Lean<br>44 Asthma Ov<br>39 Asthma Ob	-	7/13 Asthma Lean<br>10/4 Asthma Ov<br>9/6 Asthma Ob	79 Asthma Lean<br>73 Asthma Ov<br>66 Asthma Ob	94 Asthma Lean<br>93 Asthma Ov<br>84 Asthma Ob	71 Asthma Lean<br>65 Asthma Ov<br>64 Asthma Ob	Mixed	SG
The impact of abdominal adiposity measured by sonography on the pulmonary function of pre-menopausal females	Zied Rasslan	2015	Brazil	Cross Sectional	1	25 Lean (BMI 18.5-24.9)<br>28 Ov (BMI 25-29.9)<br>27 Ob (BMI≥30)	31.2 ± 7.11 Lean<br>31.9 ± 6.85 Ov<br>32.9 ± 7.25 Ob	22.4 ± 1.57 Lean<br>27.8 ± 1.25 Ov<br>33.3 ± 2.18 Ob	0/25 Lean<br>0/28 Ov<br>0/27 Ob	99.4 ± 9.46 Lean<br>101.5 ± 12.1 Ov<br>99 ± 8.71 Ob	102.5 ± 11.6 Lean<br>104.2 ± 12.2 Ov<br>100.6 ± 10 Ob	84 ± 4 Lean<br>83 ± 4 Ov<br>85 ± 5 Ob	Never	RL
Status of Pulmonary function in Indian young overweight male individuals	Jnaneshwar Shenoy	2011	India	Cross Sectional	1	48 Lean (BMI 18-25)	20.8 ± 2.62 Lean	21.62 ± 1.52 Lean	48/0 Lean	-	-	84.99 ± 4.58 Lean	Never	RL
A comparative study of FVC, FEV1, FEV1/FVC ratio before and after cycling in young obese and non-obese women	A.J. Divya	2022	India	Cross sectional	1	50 Lean (BMI 18-22.9)	-	19.82 ± 1.39 Lean	0/50 Lean	98.4 ± 1.07 Lean	97.8 ± 2.46 Lean	85.11 ± 3.63 Lean	-	RL
A new approach for the detection of obesity-related airway obstruction in lung-healthy individuals	Rudolf A. Jorres	2022	Germany	Cross sectional	1	23 Lean (BMI<25)<br>66 Ob (BMI≥30)	36.3 ± 14.4 Lean<br>49.85 ± 13.24 Ob	21.4 ± 1.9 Lean<br>37.73 ± 7.04 Ob	3/20 Lean<br>18/48 Ob	93.7 ± 8.5 Lean<br>89.1 ± 14.46 Ob	94.7 ± 7.6 Lean<br>90.38 ± 12.35 Ob	82 ± 5.7 Lean<br>78.57 ± 6.2 Ob	Mixed	RL
A Study of Correlation of Pulmonary Function Tests and Body Mass Index in the MBBS Students and Health Care Workers of Bhagwan Mahavir Institute of Medical Sciences, Pawapuri	Paritosh Kumar	2022	India	Cross sectional	1	61 Ov (BMI 25-29.9)<br>17 Ob (BMI≥30)	-	-	Both	-	-	87.47 ± 6.14 Ov<br>86.27 ± 4.22 Ob	-	RL
A Study on Pulmonary Function Tests In Type 2 Diabetes Mellitus Patients- A Case Control Study From South India	D Vasantha Kalyani	2022	India	Case Control	1	25 T2DM Only (BMI<30)	-	-	15/10 T2DM Only	88.68 ± 15.73 T2DM Only	82.4 ± 12.75 T2DM Only	106.52 ± 6.35 T2DM Only	Never	RL
Allergic and non-allergic asthma phenotypes and exposure to air pollution	Busra Pekince	2022	Turkey	Case Control	1	22 Asthma Lean (BMI<24)	-	-	Both	88.81 ± 10.97 Asthma Lean	-	87.86 ± 7.75 Asthma Lean	Never	RL
Asthma phenotype: Clinical, physiological, and biochemical profiles of North Indian patients	R Naveen Vennilavan	2022	India	Cross Sectional observational study	1	90 Asthma Lean (BMI<25)	39.63 ± 14.38 Asthma Lean	21.21 ± 2.61 Asthma Lean	Both	74.93 ± 18.25 Asthma Lean	-	-	Never	RL
Body mass index increase: a risk factor for forced expiratory volume in 1 s decline for overweight and obese adults with asthma	Nicolás Bermúdez Barón	2022	Sweden	Cohort	5	485 Asthma Lean (BMI 18.5–25)<br>327 Asthma Ov (BMI 25-30)<br>133 Asthma Ob (BMI≥30)	37.7 ± 11.4 Asthma Lean<br>43.3 ± 11.2 Asthma Ov<br>43.3 ± 11.2 Asthma Ob	22.6 ± 1.7 Asthma Lean<br>27.2 ± 1.4 Asthma Ov<br>33.0 ± 3.0 Asthma Ob	182/303 Asthma Lean<br>182/145 Asthma Ov<br>55/78 Asthma Ob	90.4 ± 13.7 Asthma Lean<br>87.2 ± 13.7 Asthma Ov<br>85.1 ± 13.6 Asthma Ob	89.0 ± 11.5 Asthma Lean<br>86.3 ± 11.7 Asthma Ov<br>82.6 ± 11.7 Asthma Ob	81.2 ± 7.6 Asthma Lean<br>80 ± 7.7 Asthma Ov<br>81.6 ± 6.4 Asthma Ob	Mixed	RL
Comparison of FEV1/FVC in Type-2 Diabetes Mellitus Patients and Healthy Individuals	Sanjeev Kumar Yadav	2022	India	Cross sectional	2	216 T2DM Only (Mixed BMI)	-	-	141/ 75 T2DM Only	70.7 T2DM Only	71.61 T2DM Only	-	-	RL
Disorders of Pulmonary Function in Type 2 Diabetes Mellitus Patients With Different Types of Oral Hypoglycemic Medications: Metformin, Metformin Plus Thiazolidinedione and Metformin plus Sulfonylurea	Rua A Tariq Abdulsaied	2022	Iraq	Cross sectional	1	160 T2DM Only (BMI<40)	54.57 ± 7.85 T2DM Only	28.69 ± 4.61 T2DM Only	84/76 T2DM Only	-	-	89.61 ± 8.68 T2DM Only	Never	RL
Down-regulated surfactant protein B in obese asthmatics	Thi Bich Tra Cao	2022	Republic of Korea	Cross sectional	1	83 Asthma Lean (BMI 18.5-24.9)<br>36 Asthma Ov (BMI 25-29.9)<br>10 Asthma Ob (BMI≥30)	49.39 ± 14.54 Asthma Lean<br>54.50 ± 14.37 Asthma Ov<br>40.90 ± 14.61 Asthma Ob	21.85 ± 1.65 Asthma Lean<br>26.45 ± 1.22 Asthma Ov<br>32.59 ± 4.40 Asthma Ob	24/59 Asthma Lean<br>12/24 Asthma Ov<br>7/3 Asthma Ob	-	-	82.33 ± 9.28 Asthma Lean<br>85.06 ± 7.27 Asthma Ov<br>81.41 ± 7.31 Asthma Ob	-	RL
[illegible] predictor of pulmonary microangiopathy and its association with extra pulmonary microangiopathy in patients with type II diabetes mellitus	Sridatta Gurudatta Pawar	2022	India	Cross sectional	1	80 T2DM Only (Mixed BMI)	51.11 ± 10.15 T2DM Only	25.14 ± 3.61 T2DM Only	43/37 T2DM Only	69.77 ± 14.94 T2DM Only	75.98 ± 17.39 T2DM Only	-	Never	RL
Effect of age, gender, and body mass index on peak expiratory flow rate and other pulmonary function tests in healthy individuals in the age group 18-60 years	Komal Mankar	2022	India	Cross sectional	1	105 Lean (BMI 18-24.9)<br>40 Ov (BMI 25-29.9)<br>22 Ob (BMI≥30)	-	-	Both	96.6 ± 20.7 Lean<br>86.7 ± 24.2 Ov<br>82.9 ± 24.3 Ob	86.2 ± 18.9 Lean<br>79.8 ± 18.5 Ov<br>72.4 ± 23.0 Ob	-	Unknown Lean<br>Unknown Ov<br>Never Ob	RL
Increased airway resistance can be related to the decrease in the functional capacity in obese women	Larissa Perossi	2022	Brazil	Cross sectional	1	37 Ob (BMI≥40)	-	-	0/37 Ob	93.72 ± 14.14 Ob	95.11 ± 12.80 Ob	-	Never	RL
Liraglutide Improves Forced Vital Capacity in Individuals With Type 2 Diabetes: Data From the Randomized Crossover LIRALUNG Study.	Carolina Lopez-Cano	2022	Spain	Randomised Controlled Trial	5	76 T2DM Ob (BMI≥30)	58.6 ± 7.5 T2DM Ob	34.8 ± 4.1 T2DM Ob	46/30 T2DM Ob	77.5 ± 10.8 T2DM Ob	81.1 ± 17.9 T2DM Ob	-	Mixed	RL
Obesity in women with asthma: Baseline disadvantage plus greater small-airway responsiveness	Arnaud Bourdin	2023	France	Cohort	1	13 Asthma Ob (BMI≥30)	-	36.27 ± 5.81 Asthma Ob	0/13 Asthma Ob	73.11 ± 13.44 Asthma Ob	84.57 ± 11.07 Asthma Ob	71.89 ± 7.89 Asthma Ob	Mixed	RL
Physical activity levels in asthma: relationship with disease severity, body mass index and novel accelerometer-derived metrics	Helen Clare Ricketts	2022	UK	Cross sectional	Multiple	25 Asthma Lean (BMI<25)	-	-	8/17 Asthma Lean	95.1 Asthma Lean		73.7 Asthma Lean	Mixed	RL
Study of Pulmonary Function Tests in Diabetic Nephropathy	Ch Ashrith	2022	India	Cross sectional	1	100 T2DM Only (Mixed BMI)	59.88 ± 9.65 T2DM Only	24.87 ± 3.6 T2DM Only	Both	66.76 ± 16.54 T2DM Only	61.42 ± 16.03 T2DM Only	89.18 ± 6.6 T2DM Only	Never	RL

Papers removed

	Title	Reason
1	A comprehensive analysis of factors related to lung function in older adults: Cross-sectional findings from the Canadian Longitudinal Study on Aging	Categories not clearly separated for analysis
2	A history of diabetes but not hyperglycaemia during exacerbation of obstructive lung disease has impact on long-term mortality: a prospective, observational cohort study	No FEV1 or FVC data (mean ± SD)
3	A Low Lean-to-Fat Ratio Reduces the Risk of Acute Exacerbation of Chronic Obstructive Pulmonary Disease in Patients with a Normal or Low Body Mass Index	Obesity not measured by BMI
4	A Strong Graded Relationship between Level of Obesity and COPD: Findings from a National Population-Based Study of Lifelong Nonsmokers	No FEV1 or FVC data (mean ± SD)
5	A study of metabolic syndrome in chronic obstructive pulmonary disease patients attending out-patient department of a medical college	No FEV1 or FVC data (mean ± SD)
6	Analysis of prevalence and prognosis of type 2 diabetes mellitus in patients with acute exacerbation of COPD	No FEV1 or FVC data (mean ± SD)
7	Anthropometric status of individuals with COPD in the city of São Paulo, Brazil, over time - analysis of a population-based study	Categories not clearly separated for analysis
8	Assessment of Glycemic Control in Veterans With Chronic Obstructive Pulmonary Disease and Type 2 Diabetes Mellitus on Inhaled Corticosteroid Therapy	No FEV1 or FVC data (mean ± SD)
9	Association between adiposity measures and COPD risk in Chinese adults	No FEV1 or FVC data (mean ± SD)
10	Association between asthma/chronic obstructive pulmonary disease overlap syndrome and healthcare utilization among the US adult population	No FEV1 or FVC data (mean ± SD)
11	Association between Comorbidities and Preserved Ratio Impaired Spirometry: Using the Korean National Health and Nutrition Examination Survey IV–VI	Categories not clearly separated for analysis
12	Association Between Forced Expiratory Volume in one Second and Glycated Hemoglobin Values in Patients With Chronic Obstructive Pulmonary Disease	Categories not clearly separated for analysis
13	Association between insulin resistance and BMI with FEV1 in non- hypoxemic COPD out- patients	Categories not clearly separated for analysis
14	Association between prevalence of obstructive lung disease and obesity: results from The Vermont Diabetes Information System	No FEV1 or FVC data (mean ± SD)
15	Association of restrictive ventilatory dysfunction with insulin resistance and type 2 diabetes in Koreans	Categories not clearly separated for analysis
16	Association of restrictive ventilatory dysfunction with the development of prediabetes and type 2 diabetes in Koreans	Categories not clearly separated for analysis
17	Body mass index, respiratory conditions, asthma, and chronic obstructive pulmonary disease	No FEV1 or FVC data (mean ± SD)
18	Body size and physical activity in relation to incidence of chronic obstructive pulmonary disease	No FEV1 or FVC data (mean ± SD)
19	Characteristics and outcomes of diabetic patients with acute exacerbation of COPD	No FEV1 or FVC data (mean ± SD)
20	Chronic obstructive pulmonary disease and metabolic syndrome: a nationwide survey in Korea	No FEV1 or FVC data (mean ± SD)
21	Chronic obstructive pulmonary disease: a risk factor for type 2 diabetes: a nationwide population-based study	No FEV1 or FVC data (mean ± SD)
22	Clinical and sociodemographic characteristics of women diagnosed with chronic obstructive pulmonary disease (COPD) in Spain: ECME study	Paper not in English
23	Clinical impact of obesity on respiratory diseases: A real-life study	No FEV1 or FVC data (mean ± SD)
24	Clinical significance of high-mobility group box-1 (HMGB1) in subjects with type 2 diabetes mellitus (T2DM) combined with chronic obstructive pulmonary disease (COPD).	No FEV1 or FVC data (mean ± SD)
25	Co-Morbidity, Body Mass Index and Quality of Life in COPD Using the Clinical COPD Questionnaire	No FEV1 or FVC data (mean ± SD)
26	Comorbidities and Burden of COPD: A Population Based Case-Control Study	No FEV1 or FVC data (mean ± SD)
27	Comorbidities in chronic obstructive pulmonary disease: Results ofa national multicenter research project	Not confirmed COPD, asthma or T2DM

28	Comorbidities in obstructive lung disease in Korea: data from the fourth and fifth Korean National Health and Nutrition Examination Survey	No FEV1 or FVC data (mean ± SD)
29	Comorbidities of COPD in Bulgarian Patients - Prevalence and Association with Severity and Inflammation.	No FEV1 or FVC data (mean ± SD)
30	Comorbidity associated with obesity in alarge population: The APNA study	No FEV1 or FVC data (mean ± SD)
31	Comorbidity between chronic obstructive pulmonary disease and type2 diabetes: A nation-wide cohort twin study	No FEV1 or FVC data (mean ± SD)
32	Comorbidity in patients with chronic obstructive pulmonary disease in family practice: a cross sectional study	No FEV1 or FVC data (mean ± SD)
33	Distribution of body mass index among subjects with COPD in the Middle East and North Africa region: Data from the BREATHE study	No FEV1 or FVC data (mean ± SD)
34	Ectopic fat accumulation in patients with COPD: an ECLIPSE substudy	Obesity not measured by BMI
35	Excessive visceral fat accumulation in advanced chronic obstructive pulmonary disease	Not journal article
36	Exploring the impact of chronic obstructive pulmonary disease (COPD) on diabetes control in diabetes patients: a prospective observational study in general practice	No FEV1 or FVC data (mean ± SD)
37	Extreme Values of Hemoglobin A1c Are Associated With Increased Risks of Chronic Obstructive Pulmonary Disease in Patients With Type 2 Diabetes	No FEV1 or FVC data (mean ± SD)
38	Glucose Levels in Patients With Acute Respiratory Failure Requiring Mechanical Ventilation	No FEV1 or FVC data (mean ± SD)
39	Glycated hemoglobin A1c-based adjusted glycemic variables in patients with diabetes presenting with acute exacerbation of chronic obstructive pulmonary disease	No FEV1 or FVC data (mean ± SD)
40	Identification and prospective validation of clinically relevant chronic obstructive pulmonary disease (COPD) subtypes	Categories not clearly separated for analysis
41	Impact of overweight and obesity on acute exacerbations of COPD – subgroup analysis of the Taiwan Obstructive Lung Disease cohort	No FEV1 or FVC data (mean ± SD)
42	Incidence of severe exacerbation in patients diagnosed with diabetes and chronic obstructive pulmonary disease: Cohort study	Paper not in English
43	Incidence of type II diabetes in chronic obstructive pulmonary disease: a nested case–control study	No FEV1 or FVC data (mean ± SD)
44	Increased risk of respiratory diseases in adults withType 1 and Type 2 diabetes	No FEV1 or FVC data (mean ± SD)
45	Individual approach to the treatment of obese copd patients can reduce anthropometric indicators, the level of systemic inflammation and improve the quality of life	Categories not clearly separated for analysis
46	Insulin resistance may contribute to vascular dysfunction in patients with chronic obstructive pulmonary disease	Categories not clearly separated for analysis
47	Metabolic syndrome in patients with chronic obstructive pulmonary disease	Categories not clearly separated for analysis
48	Obesity in Bulgarian patients with chronic obstructive pulmonary disease	Categories not clearly separated for analysis
49	Obesity in COPD: Comorbidities with Practical Consequences?	Categories not clearly separated for analysis
50	Pathophysiological Correlation between Diabetes Mellitus Type-II and Chronic Obstructive Pulmonary Diseases	No FEV1 or FVC data (mean ± SD)
51	Patterns of Body Composition Relating to Chronic Respiratory Diseases Among Adults in Four Resource-Poor Settings in Peru	Not confirmed COPD, asthma or T2DM
52	Population-based cohort study suggesting a significantly increased risk of developing chronic obstructive pulmonary disease in people with type 2 diabetes mellitus	No FEV1 or FVC data (mean ± SD)
53	Prevalence of metabolic syndrome in copd in rural population of developing country- a cross-sectional study	No FEV1 or FVC data (mean ± SD)
54	Prevalence of metabolic syndrome in patients with chronic obstructive pulmonary disease: An observational study in South Indians	No FEV1 or FVC data (mean ± SD)
55	Relationship between glycemic control and chronic obstructive pulmonary disease in patients with type 2 diabetes: A nested case-control study	No FEV1 or FVC data (mean ± SD)
56	The Association between BMI and COPD: The Results of Two Population-based Studies in Guangzhou, China	Categories not clearly separated for analysis
57	The Prevalence of Diabetes Mellitus in COPD Patients with Severe and Very Severe Stage of the Disease	Categories not clearly separated for analysis

58	Type 2 diabetes: A protective factor for COPD?	No FEV1 or FVC data (mean ± SD)
59	Uncovering Metabolic Syndrome among Chronic Obstructive Pulmonary Disease Patients in a Tertiary Care Hospital, India	No FEV1 or FVC data (mean ± SD)
60	Visit-to-visit glycemic variability is a strong predictor of chronic obstructive pulmonary disease in patients with type 2 diabetes mellitus: Competing risk analysis using a national cohort from the Taiwan diabetes study	No FEV1 or FVC data (mean ± SD)
61	A prospective study of the impact of diabetes mellitus on restrictive and obstructive lung function impairment: The Saku study	Categories not clearly separated for analysis
62	Association between obesity and chronic obstructive pulmonary disease in Moroccan adults: Evidence from the BOLD study	No FEV1 or FVC data (mean ± SD)
63	Diabetes mellitus among outpatients with COPD attending a university hospital	No FEV1 or FVC data (mean ± SD)
64	Lung function and metabolic syndrome: Findings of National Health and Nutrition Examination Survey 2007–2010	Categories not clearly separated for analysis
65	Only severe COPD is associated with being underweight: results from a population survey	Not confirmed COPD, asthma or T2DM
66	The Association of Body Mass Index with Airway Obstruction in Non-Asthmatics: Implications for the Inaccurate Differential Diagnosis of Asthma in Obesity	Categories not clearly separated for analysis
67	Waist Circumference and Spirometric Measurements in Chronic Obstructive Pulmonary Disease	Obesity not measured by BMI
68	Study the relation between body mass index,waist circumference and spirometry in COPD patients	Obesity not measured by BMI
69	Association between restrictive pulmonary disease and type 2 diabetes in Koreans: A cross-sectional study	Categories not clearly separated for analysis
70	The Association Between Lung Function and Type 2 Diabetes in Koreans	Categories not clearly separated for analysis
71	Prevalence of metabolic syndrome in COPD patients and its consequences	Not confirmed COPD, asthma or T2DM
72	High body mass index and risk of exacerbations and pneumonias in individuals with chronic obstructive pulmonary disease: observational and genetic risk estimates from the Copenhagen General Population Study	Categories not clearly separated for analysis
73	A blinded evaluation of the efficacy and safety of glycopyrronium, a once-daily long-acting muscarinic antagonist, versus tiotropium, in patients with COPD: The GLOW5 study	No FEV1 or FVC data (mean ± SD)
74	A comparative study of impact of obesity on maximum voluntary ventilation in young adult women	No FEV1 or FVC data (mean ± SD)
75	A Dyadic Growth Modeling Approach for Examining Associations Between Weight Gain and Lung Function Decline	Categories not clearly separated for analysis
76	Abdominal adiposity is an early marker of pulmonary function impairment: Findings from a Mediterranean Italian female cohort	Categories not clearly separated for analysis
77	Adiposity markers and lung function in smokers: A cross-sectional study in a Mediterranean population	Categories not clearly separated for analysis
78	Adiposity: determinant of peak expiratory flow rate in young Indian adults male	No FEV1 or FVC data (mean ± SD)
79	Anthropometric determinants of peak expiratory flow rate and forced expiratory volume in first second in healthy young adults of eastern India	Categories not clearly separated for analysis
80	Association between insulin resistance and lung function trajectory over 4 years in South Korea: community-based prospective cohort	Not confirmed COPD, asthma or T2DM
81	Association between lung capacity and abnormal glucose metabolism: Findings from China and Australia	No FEV1 or FVC data (mean ± SD)
82	Association between lung capacity measurements and abnormal glucose metabolism: Findings from the Crossroads study	No FEV1 or FVC data (mean ± SD)
83	Association between metabolic syndrome and rate of lung function decline: a longitudinal analysis	Categories not clearly separated for analysis
84	Association of adiposity with pulmonary function in older Chinese: Guangzhou Biobank Cohort Study	Obesity not measured by BMI

85	Association of body mass index with lung function parameters in non-asthmatics identified by spirometric protocols	Categories not clearly separated for analysis
86	Association of Pulmonary Functions and HbA1c in Diabetics	Categories not clearly separated for analysis
87	Body mass index and weight change are associated with adult lung function trajectories: the prospective ECRHS study	Categories not clearly separated for analysis
88	Cardiorespiratory fitness, pulmonary function and C-reactive protein levels in nonsmoking individuals with diabetes	Categories not clearly separated for analysis
89	Comparative study of forced expiratory flow and peak expiratory flow in males with and without type-2 diabetes mellitus	No FEV1 or FVC data (mean ± SD)
90	Comparison of body composition parameters in the study of the association between body composition and pulmonary function	Under age 18
91	Correlation of gender and body mass index with pulmonary function tests in medical and paramedical students of Muzaffarnagar Medical College	Categories not clearly separated for analysis
92	Decline in lung function rather than baseline lung function is associated with the development of metabolic syndrome: A sixyear longitudinal study	Not confirmed COPD, asthma or T2DM
93	Diabetes, Impaired Glucose Tolerance, and Metabolic Biomarkers in Individuals with Normal Glucose Tolerance are Inversely Associated with Lung Function: The Jackson Heart Study	Categories not clearly separated for analysis
94	Does BMI affect lung functions?	Full text not available
95	Effect of gender on the peak expiratory flow rate in diabetic population: A pilot study	No FEV1 or FVC data (mean ± SD)
96	Assessment of pulmonary functions in overweight adults - A case control study	Not journal article
97	Effect of phrenic neuropathy on forced expiratory flow rates in patients with type 2 diabetes mellitus	Not journal article
98	Effects of fat distribution on lung function in young adults	No FEV1 or FVC data (mean ± SD)
99	Glycemic control and pulmonary function tests in type 2 diabetes mellitus patients: Do they correlate?	Not journal article
100	Glycemic disorders and their impact on lung function. Cross-sectional study	Categories not clearly separated for analysis
101	Hispanics/Latinos With Type 2 Diabetes Have Functional and Symptomatic Pulmonary Impairment Mirroring Kidney Microangiopathy: Findings From the Hispanic Community Health Study/Study of Latinos (HCHS/SOL)	Categories not clearly separated for analysis
102	Impact of diabetes mellitus and its control on pulmonary functions and cardiopulmonary exercise tests	Categories not clearly separated for analysis
103	Impact of diabetes mellitus on functional exercise capacity and pulmonary functions in patients with diabetes and healthy persons	Categories not clearly separated for analysis
104	Low Lung Function and Risk of Type 2 Diabetes in Japanese Men: The Toranomon Hospital Health Management Center Study 9 (TOPICS 9)	Not confirmed COPD, asthma or T2DM
105	Lung: Another Victim of the Silent killer Diabetes- A Cross-sectional Study	No FEV1 or FVC data (mean ± SD)
106	Metabolic health is more closely associated with decrease in lung function than obesity	Not confirmed COPD, asthma or T2DM
107	Non-linear association between diabetes mellitus and pulmonary function: a population-based study	Categories not clearly separated for analysis
108	Nonlinear relationship between visceral adiposity index and lung function: a population-based study	Obesity not measured by BMI

109	Obesity Duration Is Associated to Pulmonary Function Impairment in Obese Subjects	Under age 18
110	Obesity in adults is associated with reduced lung function in metabolic syndrome and diabetes: The strong heart study	Categories not clearly separated for analysis
111	Obesity parameters in relation to lung function levels in a large Chinese rural adult population	Categories not clearly separated for analysis
112	Pericardial Fat Is Associated With Impaired Lung Function and a Restrictive Lung Pattern in Adults	Categories not clearly separated for analysis
113	Pulmonary function abnormalities and Type 2 Diabetes Mellitus – a cross-sectional study	No FEV1 or FVC data (mean ± SD)
114	Restrictive Pulmonary Disease in Diabetes Mellitus Type II Patients	No FEV1 or FVC data (mean ± SD)
115	The effects of truncal adiposity in forced spirometry: Sex differences	Obesity not measured by BMI
116	The Impact of Body Mass Index on the Expiratory Reserve Volume	No FEV1 or FVC data (mean ± SD)
117	The impact of body mass index, central obesity and physical activity on lung function: results of the EpiHealth study	No FEV1 or FVC data (mean ± SD)
118	The longitudinal association between changes in lung function and changes in abdominal visceral obesity in Korean non-smokers	Categories not clearly separated for analysis
119	The relation between insulin resistance and lung function: a cross sectional study	Not confirmed COPD, asthma or T2DM
120	The Relationship between Insulin Resistance and Pulmonary Functions in Morbidly Obese Patients	Not confirmed COPD, asthma or T2DM
121	The temporal relationship between poor lung function and the risk of diabetes	No FEV1 or FVC data (mean ± SD)
122	Visceral adipose tissue level, as estimated by the bioimpedance analysis method, is associated with impaired lung function	Categories not clearly separated for analysis
123	Visceral adiposity index is associated with lung function impairment: a population-based study	Obesity not measured by BMI
124	Association and correlation between cardiorespiratory fitness, bmi, musculo-skeletal and handgrip strength among young adult student girl population in Sullia, Karnataka, India	Categories not clearly separated for analysis
125	Association of Pulmonary Function Decline over Time with Longitudinal Change of Glycated Hemoglobin in Participants without Diabetes Mellitus	Categories not clearly separated for analysis
126	Effect of obesity on pulmonary function	Full text not available
127	Study of lung function tests in type II Diabetes Mellitus in west Bengal	Full text not available
128	Type 2 Diabetes is Associated with Lower Cardiorespiratory Fitness Independent of Pulmonary Function in Severe Obesity	Full text not available
129	Effect of increasing weight on spirometry in young healthy adult female: A study in West Bengal	Full text not available
130	Effect of Type 2 Diabetes Mellitus and Diabetic Medication on Pulmonary Function	Full text not available
131	Body composition from 18 to 22 years and pulmonary function at 22 years-1993 Pelotas Birth Cohort	Categories not clearly separated for analysis
132	The effect of obesity on spirometry tests among healthy non-smoking adults	Categories not clearly separated for analysis
133	COPD patients' body composition and its impact on lung function	Categories not clearly separated for analysis
134	Overweight and obesity may lead to under-diagnosis of airflow limitation: findings from the Copenhagen City Heart Study.	Not confirmed COPD, asthma or T2DM
135	Association of obesity and asthma in Korea	Not journal article
136	Are a high body mass index or large waist circumference associated with pulmonary dysfunction?	Under age 18
137	Lung function impairment in women aged over 40 years: The critical role of abdominal obesity	Categories not clearly separated for analysis

138	Leptin is inversely associated with lung function in African Americans, independent of adiposity: the Jackson Heart Study.	No FEV1 or FVC data (mean ± SD)
139	Exploring the obesity-asthma link: do all types of adiposity increase the risk of asthma?.	Obesity not measured by BMI
140	The relationship between insulin resistance and pulmonary function in overweight or obese US adults with asthma	Full text not available
141	Obesity is associated with an increased respiratory workload during exercise	Not journal article
142	Effect of glycaemic control on pulmonary function: Data from a 3- months interventional study	Not journal article
143	Effect of exercise on pulmonary function tests in obese Malaysian patients	Obesity not measured by BMI
144	Assessment of change in pulmonary function parameters in type 2 diabetics as compared to non diabetic subjects	Not journal article
145	Accelerometer-based physical activity in daily life is not positively associated with better pulmonary function in adult smokers without airflow obstruction	Not journal article
146	Predictors of accelerated FEV1 decline in adults with airflow limitation-Findings from the Health2006 cohort.	Categories not clearly separated for analysis
147	Change of respiratory functions, the STOP-Bang questionnaire, and Epworth sleepiness scale after bariatric surgery.	Not confirmed COPD, asthma or T2DM
148	Lung function as a predictor of incident type 2 diabetes in community-dwelling adults: A longitudinal finding over 12 years from the Korean Genome and Epidemiology Study.	Categories not clearly separated for analysis
149	Body Mass Index in Adult Asthmatic Patients.	No FEV1 or FVC data (mean ± SD)
150	The impact of therapy on the risk of asthma in type 2 diabetes.	No FEV1 or FVC data (mean ± SD)
151	Effect of Intentional Weight Loss on Mortality Biomarkers in Older Adults With Obesity.	Categories not clearly separated for analysis
152	Poor asthma control in older adults is linked to chronic exposure to traffic pollutants and obesity	No FEV1 or FVC data (mean ± SD)
153	Respiratory function in superobese patients before and after bariatric surgery- a randomised controlled trial	Not confirmed COPD, asthma or T2DM
154	Weight loss and pulmonary function changes in middle age adults: The cardia study	Not confirmed COPD, asthma or T2DM
155	Association between glycaemic control and impaired lung function in Japanese adultsAssociation of Glycemic Status With Impaired Lung Function Among Recipients of a Health Screening Program: A Cross-Sectional Study in Japanese Adults	Categories not clearly separated for analysis
156	Burden of reduced FEV1 (<80% of predicted) and doctor diagnosed asthma and their association with smoking and BMI among urban adult population in Barrackpore, West Bengal (India)	Full text not available
157	Clinical characteristics, airway inflammation, and adipocytokines in overweight and obese asthmatics	Not journal article
158	Correlation of body mass index with maximum respiratory pressures	Full text not available
159	Discordance Between Forced Vital Capacity And Slow Vital Capacity In Overweight And Obese People Leads To Underdiagnosis Of Obstructive Airway Disease	Full text not available
160	Effect of obesity and metabolic syndrome on hypoxic vasodilation	Not confirmed COPD, asthma or T2DM
161	Gender Differentially Contributes To Airway Hyperresponsiveness In Adult Asthmatics	Full text not available
162	Improved Air Quality and Attenuated Lung Function Decline: Modification by Obesity in the SAPALDIA Cohort	Categories not clearly separated for analysis
163	Improvement Of Lung And Cardiac Function By Weight Loss In Obese Patients	Not journal article
164	Influence of asthma, smoking, and obesity on lung function parameters in the us adult population: NHANES 2007-2012	Full text not available

165	Influence of obesity and overweight in lung function in adult asthmatic patients	Not journal article
166	Low FEV1, smoking history, and obesity are factors associated with oxygen saturation decrease in an adult population cohort	Categories not clearly separated for analysis
167	Low Vital Capacity was Found to be Associated with Incident Diabetes in Health Screening Population of Japanese Women	Categories not clearly separated for analysis
168	Obesity and asthma control	Not journal article
169	Obesity and Asthma: association or epiphenomenon?	No FEV1 or FVC data (mean ± SD)
170	Obesity, Metabolic Syndrome And Asthma	Not journal article
171	Overweight and obesity as risk factors for impaired lung function in patients with asthma: A real-life experience.	Not journal article
172	Overweight/obesity and adult onset asthma in Chinese population	Not journal article
173	The effect of obesity on dyspnea, exercise capacity, walk work and workload in patients with COPD.	Not confirmed COPD, asthma or T2DM
174	Overnight Changes in Lung Function of Obese Patients with Obstructive Sleep Apnoea.	Not confirmed COPD, asthma or T2DM
175	Severe asthma in Kuwait population: Phenotype-based approach.	No FEV1 or FVC data (mean ± SD)
176	Functional significance of 8-isoprostanes in sinonasal disease and asthma.	Full text not available
177	Evaluation of a Multidisciplinary Disease Management Program to Achieve Asthma Control in Seven Safety Net Hospitals in Louisiana.	Full text not available
178	Glycemic management is inversely related to skeletal muscle microvascular endothelial function in patients with type 2 diabetes.	No FEV1 or FVC data (mean ± SD)
179	Association between HOMA-IR and Lung Function in Korean Young Adults based on the Korea National Health and Nutrition Examination Survey.	No FEV1 or FVC data (mean ± SD)
180	IL-26 in the induced sputum is associated with the level of systemic inflammation, lung functions and body weight in COPD patients.	No FEV1 or FVC data (mean ± SD)
181	Interactive effects of adiposity and insulin resistance on the impaired lung function in asthmatic adults: cross-sectional analysis of NHANES data.	Categories not clearly separated for analysis
182	Lung function measurements in the prediabetes stage: data from the ILERVAS Project.	Categories not clearly separated for analysis
183	Influence of Obesity on Work Ability, Respiratory Symptoms, and Lung Function in Adults with Asthma.	Full text not available
184	The association of body mass index, weight gain and central obesity with activity-related breathlessness: the Swedish Cardiopulmonary Bioimage Study.	Full text not available
185	Association between air flow limitation and body composition in young adults	Categories not clearly separated for analysis
186	Causal Effects of Body Mass Index on Airflow Obstruction and Forced Mid-Expiratory Flow: A Mendelian Randomization Study Taking Interactions and Age-Specific Instruments Into Consideration Toward a Life Course Perspective	Categories not clearly separated for analysis
187	Effect of bariatric surgery on asthma control, lung function and bronchial and systemic inflammation in morbidly obese subjects with asthma.	No FEV1 or FVC data (mean ± SD)
188	Factors reducing omalizumab response in severe asthma	Categories not clearly separated for analysis
189	Influence of weight loss on pulmonary function and levels of adipokines among asthmatic individuals with obesity: One-year follow-up	No FEV1 or FVC data (mean ± SD)
190	Long-term observational study on the impact of GLP-1R agonists on lung function in diabetic patients	Categories not clearly separated for analysis
191	Genetic correlation of lung function with anthropometric measures in the busselton health study	Full text not available
192	Obesity-Related Indices Are Associated with Longitudinal Changes in Lung Function: A Large Taiwanese Population Follow-Up Study	Categories not clearly separated for analysis
193	Opportunistic screening for COPD in primary care: a pooled analysis of 6,710 symptomatic smokers and ex-smokers	Categories not clearly separated for analysis

194	Predictors in routine practice of 6-min walking distance and oxygen desaturation in patients with COPD: impact of comorbidities	Categories not clearly separated for analysis
195	Regular Physical Activity Levels and Incidence of Restrictive Spirometry Pattern: A Longitudinal Analysis of 2 Population-Based Cohorts	Categories not clearly separated for analysis
196	Relationship between metabolic syndrome and pulmonary function in workers with respiratory dust exposure in Iran	Not confirmed COPD, asthma or T2DM
197	Relationship between pulmonary function and albuminuria in type 2 diabetic patients with preserved renal function	Obesity not measured by BMI
198	The association between anthropometric measures and lung function in a population-based study of Canadian adults	Categories not clearly separated for analysis
199	The association of two different measures of body habitus with lung function: a population-based study	Categories not clearly separated for analysis
200	The multi-ethnic global lung initiative 2012 (GLI-2012) norms reflect contemporary adult's Algerian spirometry	Categories not clearly separated for analysis
201	The Relationship between Diabetes Mellitus and Respiratory Function in Patients Eligible for Coronary Artery Bypass Grafting	Categories not clearly separated for analysis
202	Central obesity and other factors associated with uncontrolled asthma in women	Categories not clearly separated for analysis
203	Smoking and obesity increase airway hyperesponsiveness risk in the elderly	Categories not clearly separated for analysis
204	Distal airway impairment in obese normoreactive women	No FEV1 or FVC data (mean ± SD)
205	Alterations in lung functions based on BMI and body fat % among obese indian population at National Capital Region	Categories not clearly separated for analysis
206	The effect of obesity or overweight on airway hyperresponsiveness and clinical features in patients with asthma	Data repeated from another study
207	Replication of the severe asthma research program cluster analysis in an urban population	Not journal article
208	Obesity and disease phenotypes in mild-to-moderate persistent asthma	Not journal article
209	Correlation of body mass index and waist hip ratio on pulmonary function tests	Full text not available
210	Correlation of dynamic pulmonary function tests in relation to obesity in young adults	Full text not available
211	Improvement of physical capacity, but not of pulmonary function, is sustained upon high-intensity interval training in type 2 diabetes	Full text not available
212	Change in pulmonary function over time in obese compared to healthy weight asthmatics	Not journal article
213	Benefits of omalizumab in obese patients with severe asthma	Not journal article
214	Association between low lung function and metabolic syndrome, systemic inflammation and cardiovascular event risk in healthy Korean subjects	Not journal article
215	Association between Body Mass Index (BMI) and fraction of exhaled nitric oxide (FeNO) levels in the National Health and Nutrition Examination Survey (NHANES) 2007–2010	Categories not clearly separated for analysis
216	A comparative study of FVC, FEV1, and TLC in nonsmoking Saudi students at Eastern Province, Saudi Arabia, with Caucasian reference values	Not journal article
217	Prevalence of restrictive lung function in children and adults in the general population.	No FEV1 or FVC data (mean ± SD)
218	The association of asthma duration with body mass index and Weight-Adjusted-Waist index in a nationwide study of the U.S. adults.	No FEV1 or FVC data (mean ± SD)
219	Long-term effect of asthma on the development of obesity among adults: an international cohort study, ECRHS.	No FEV1 or FVC data (mean ± SD)
220	A 4-Year Retrospective Claims Analysis of Oral Corticosteroid Use and Health Conditions in Newly Diagnosed Medicare FFS Patients with COPD	No FEV1 or FVC data (mean ± SD)
221	A Brazilian randomized study: Robotic-Assisted vs. Video-assisted lung lobectomy Outcomes (BRAVO trial)	Categories not clearly separated for analysis

222	A Comparative Study of Body Composition and Pulmonary Functions in Young Adults having Conventional Exercises, Yoga and Sedentary Lifestyle	Categories not clearly separated for analysis
223	A Cross-Sectional Study on Prescription Patterns of Short-Acting beta2-Agonists in Patients with Asthma: Results from the SABINA III Colombia Cohort	No FEV1 or FVC data (mean ± SD)
224	A Longitudinal Study of Trajectories and Factors Influencing Patient-Reported Outcomes in Chronic Obstructive Pulmonary Disease	Categories not clearly separated for analysis
225	A machine learning approach to characterize patients with asthma exacerbation attending an acute care setting	Categories not clearly separated for analysis
226	A multifaceted stewardship intervention helps curb steroid overprescribing in hospitalized patients with acute exacerbations of COPD.	No FEV1 or FVC data (mean ± SD)
227	A step-down experience in asthma treatment: a retrospective cohort study	Categories not clearly separated for analysis
228	A Study of Prevalence of Changes in FEV1 in Asthmatic Patient and Correlation with BMI	Categories not clearly separated for analysis
229	A Study on the Relationship between Serum Vitamin D Level and FEV1 in patients with Chronic Obstructive Pulmonary Disease in a Tertiary Care Hospital in India	No FEV1 or FVC data (mean ± SD)
230	A total diet replacement weight management programme for difficult-to-treat asthma associated with obesity: a randomised controlled feasibility trial	Categories not clearly separated for analysis
231	Abdominal obesity in COPD is associated with specific metabolic and functional phenotypes	Categories not clearly separated for analysis
232	Adiposity and risks of vascular and non-vascular mortality among Chinese adults with type 2 diabetes: A 10-year prospective study	No FEV1 or FVC data (mean ± SD)
233	All Types Obesity and Physical Inactivity Associated with the Risk of Activity of Daily Living Limitations Among People with Asthma	No FEV1 or FVC data (mean ± SD)
234	Analysis of Blood Pressure and Ventilation Efficiency in Different Types of Obesity Aged 40-60 Years by Cardiopulmonary Exercise Test	No FEV1 or FVC data (mean ± SD)
235	Analysis of Patients with Asthma and Mixed Granulocytic Inflammatory Pattern in Sputum	Categories not clearly separated for analysis
236	Analysis on the Risk Factors of Malnutrition in Type 2 Diabetes Mellitus Patients with Pulmonary Tuberculosis	Not confirmed COPD, asthma or T2DM
237	Are you aware "asthmatic nephropathy"? Metabolic and renal parameters in newly diagnosed untreated asthmatic patients without diabetes	No FEV1 or FVC data (mean ± SD)
238	Assessment of Cardiorespiratory and Metabolic Responses in Women with Obesity After Surgically Induced Weight Loss: Results from a Pilot Study	Categories not clearly separated for analysis
239	Association between obesity-related dyspnea in daily living, lung function and body composition analyzed by DXA: a prospective study of 130 patients	Categories not clearly separated for analysis
240	Association of BMI with pulmonary function, functional capacity, symptoms, and quality of life in ILD	Not confirmed COPD, asthma or T2DM
241	Association of Body Composition with Pulmonary Function in Ningxia: The China Northwest Cohort	No FEV1 or FVC data (mean ± SD)
242	Association of dipeptidyl peptidase-4 inhibitor use and the risk of asthma development among type 2 diabetes patients.	No FEV1 or FVC data (mean ± SD)
243	Association of general and abdominal obesity with lung function, FeNO, and blood eosinophils in adult asthmatics: Findings from NHANES 2007-2012	Categories not clearly separated for analysis
244	Association of lung function and blood glucose level: a 10-year study in China.	Categories not clearly separated for analysis
245	Association of respiratory symptoms with body mass index and occupational exposure comparing sexes and subjects with and without asthma: follow-up of a Norwegian population study (the Telemark study)	No FEV1 or FVC data (mean ± SD)

246	Association of weight change patterns across adulthood with incident asthma: a retrospective cohort study.	No FEV1 or FVC data (mean ± SD)
247	Asthma and its relationship with anthropometric markers among adults.	No FEV1 or FVC data (mean ± SD)
248	Asthma and obesity in adults	Paper not in English
249	BMI moderates the association between adverse childhood experiences and COPD.	No FEV1 or FVC data (mean ± SD)
250	Anti-Interleukin-5 Therapy Is Associated with Attenuated Lung Function Decline in Severe Eosinophilic Asthma Patients From the Belgian Severe Asthma Registry	Categories not clearly separated for analysis
251	Can we HALT obesity following lung transplant? A Dietitian- and Physiotherapy-directed pilot intervention	Not confirmed COPD, asthma or T2DM
252	Cardiovascular outcomes of metformin use in patients with type 2 diabetes and chronic obstructive pulmonary disease	No FEV1 or FVC data (mean ± SD)
253	Characterization and cluster analyses of elderly asthma in comparison with nonelderly patients with asthma in Japan	Categories not clearly separated for analysis
254	Characterization of Asthma by Age of Onset: A Multi-Database Cohort Study	No FEV1 or FVC data (mean ± SD)
255	Clinical and Lung Function Outcomes After Anti-IgE or Anti-IL5 Therapy in Severe Asthma	Categories not clearly separated for analysis
256	Clinical relevance of multiple confirmed preserved ratio impaired spirometry cases in adults	Not confirmed COPD, asthma or T2DM
257	Combined resistance and aerobic training improves lung function and mechanics and fibrotic biomarkers in overweight and obese women	Categories not clearly separated for analysis
258	Computed tomography-based body composition measures in COPD and their association with clinical outcomes: A systematic review.	Not journal article
259	Correlation of Pulmonary Function Abnormalities with Serum Lipids and Physical Parameters	Categories not clearly separated for analysis
260	Correlation of Pulmonary Function Tests with BMI in Type-2 Diabetes Mellitus	Categories not clearly separated for analysis
261	Determinants of Severe Asthma - A Long-Term Cohort Study in Northern Sweden	Categories not clearly separated for analysis
262	Differences Between Early- and Late-Onset Asthma: Role of Comorbidities in Symptom Control	Categories not clearly separated for analysis
263	Differences in Inflammatory Cytokine Profile in Obesity-Associated Asthma: Effects of Weight Loss	No FEV1 or FVC data (mean ± SD)
264	Differential effects of lung inflammation on insulin resistance in humans and mice.	No FEV1 or FVC data (mean ± SD)
265	Differential gene expression in nasal airway epithelium from overweight or obese youth with asthma	No FEV1 or FVC data (mean ± SD)
266	Dose-dependent association between inhaled corticosteroid use and risk of obesity and metabolic syndrome in asthma	No FEV1 or FVC data (mean ± SD)
267	An Online Weight Loss Intervention for People With Obesity and Poorly Controlled Asthma	No FEV1 or FVC data (mean ± SD)
268	Anxiety and body mass index affect asthma control: data from a prospective Spanish cohort	Categories not clearly separated for analysis
269	Effect of Azithromycin on Exacerbations in Asthma Patients with Obesity: Protocol for a Multi-Center, Prospective, Single-Arm Intervention Study	Not journal article
270	Effect of Diabetes mellitus in patients with acute exacerbation of the chronic obstructive pulmonary disease	No FEV1 or FVC data (mean ± SD)
271	Effect of obesity on pulmonary function test: A comparative study	No FEV1 or FVC data (mean ± SD)
272	Effects of Obstructive Sleep Apnea and Obesity on 30-Day Readmissions in Patients with Chronic Obstructive Pulmonary Disease: A Cross-Sectional Mediation Analysis	No FEV1 or FVC data (mean ± SD)
273	Empagliflozin in patients with type 2 diabetes mellitus and chronic obstructive pulmonary disease	No FEV1 or FVC data (mean ± SD)

274	Examining Risk Factors Accelerating Time-to-Chronic Obstructive Pulmonary Disease (COPD) Diagnosis among Asthma Patients.	No FEV1 or FVC data (mean ± SD)
275	Functionality of natural killer cells in obese asthma phenotypes	No FEV1 or FVC data (mean ± SD)
276	Global burden of asthma associated with high body mass index from 1990 to 2019.	No FEV1 or FVC data (mean ± SD)
277	Impact of lifetime body mass index trajectories on the incidence and persistence of adult asthma.	No FEV1 or FVC data (mean ± SD)
278	Interleukin-26 is associated with the level of systemic inflammation and lung functions in obese and non-obese moderate-to-severe asthmatic patients	No FEV1 or FVC data (mean ± SD)
279	Lifetime spirometry patterns of obstruction and restriction, and their risk factors and outcomes: a prospective cohort study	Categories not clearly separated for analysis
280	Longitudinal association between adiposity changes and lung function deterioration.	Categories not clearly separated for analysis
281	Lung function in young adulthood: differences between males and females with asthma	No FEV1 or FVC data (mean ± SD)
282	Maternal obesity, gestational weight gain, and offspring asthma and atopy	Under age 18
283	Metformin and the Development of Asthma in Patients with Type 2 Diabetes	No FEV1 or FVC data (mean ± SD)
284	Multiple Beneficial Effects of Laparoscopic Sleeve Gastrectomy for Patients with Obesity, Type 2 Diabetes Mellitus, and Restrictive Ventilatory Dysfunction.	Not confirmed COPD, asthma or T2DM
285	Novel antihyperglycaemic drugs and prevention of chronic obstructive pulmonary disease exacerbations among patients with type 2 diabetes: Population based cohort study	No FEV1 or FVC data (mean ± SD)
286	Obesity affects type 2 biomarker levels in asthma	No FEV1 or FVC data (mean ± SD)
287	Overprescription of short-acting beta2 -agonists among patients with asthma in Saudi Arabia: Results from the SABINA III cohort study.	No FEV1 or FVC data (mean ± SD)
288	Physical Activity, Exercise Capacity, and Body Composition in U.S. Veterans with Chronic Obstructive Pulmonary Disease.	No FEV1 or FVC data (mean ± SD)
289	Pioglitazone and Risk of Chronic Obstructive Pulmonary Disease in Patients with Type 2 Diabetes Mellitus: A Retrospective Cohort Study	No FEV1 or FVC data (mean ± SD)
290	Potential clinical implications of targeted spirometry for detection of COPD: A contemporary population-based cohort study	Categories not clearly separated for analysis
291	Potential for repurposing oral hypertension/diabetes drugs to decrease asthma risk in obesity	No FEV1 or FVC data (mean ± SD)
292	Preserved Ratio Impaired Spirometry, Metabolomics and the Risk of Type 2 Diabetes	No FEV1 or FVC data (mean ± SD)
293	Prevalence and Characteristics of Self-Reported Adult Asthma in Cyprus: A Population-Based Observational Study	Categories not clearly separated for analysis
294	Prevalence, risk factors, and clinical implications of preserved ratio impaired spirometry: a UK Biobank cohort analysis	No FEV1 or FVC data (mean ± SD)
295	Relationship between type 2 cytokine and inflammasome responses in obesity-associated asthma	No FEV1 or FVC data (mean ± SD)
296	Respiratory Function Correlates with Fat Mass Index and Blood Triglycerides in Institutionalized Older Individuals	Not confirmed COPD, asthma or T2DM
297	Sarcopenia is associated with cardiovascular risk in men with COPD, independent of adiposity.	Categories not clearly separated for analysis
298	Spirometry in Patients of Diabetes Mellitus	Categories not clearly separated for analysis
299	STOP: an open label crossover trial to study ICS withdrawal in patients with a combination of obesity and low-inflammatory asthma and evaluate its effect on asthma control and quality of life.	No FEV1 or FVC data (mean ± SD)

300	The effect of body mass index (BMI) on Vital Capacity:A study of corelation between Body Mass Index and Mean Forced Vital Capacity	No FEV1 or FVC data (mean ± SD)
301	The effect of metabolic health and obesity on lung function: A cross sectional study of 114,143 participants from Kangbuk Samsung Health Study	Categories not clearly separated for analysis
302	The effect of talk test-based aerobic exercise on pulmonary functions and quality of life among adults with type 2 diabetes mellitus: A randomized controlled trial	No FEV1 or FVC data (mean ± SD)
303	The influence of BMI in asthma. Which traits are due to obesity and which to asthma and obesity phenotype?	Categories not clearly separated for analysis
304	The Relationship Between BMI and Lung Function in Populations with Different Characteristics: A Cross-Sectional Study Based on the Enjoying Breathing Program in China	Not confirmed COPD, asthma or T2DM
305	The relationship between glycated hemoglobin A1c levels and exacerbation status in the patients with chronic obstructive pulmonary disease.	No FEV1 or FVC data (mean ± SD)
306	Variation Of Fev1 Andfvc With Body Mass Index In Healthy Individuals	No FEV1 or FVC data (mean ± SD)
307	Dynamic Lung Function Tests in Obese	No FEV1 or FVC data (mean ± SD)
308	Obesity and Pulmonary Function in African Americans	No FEV1 or FVC data (mean ± SD)
309	Variation Of Fev1 Andfvc With Body Mass Index In Healthy Individuals	No FEV1 or FVC data (mean ± SD)
310	Lung spirometry parameters and diffusion capacity are decreased in patients with Type 2 diabetes	No FEV1 or FVC data (mean ± SD)
311	Skeletal Muscle Adiposity and Lung Function Trajectory in the Severe Asthma Research Program.	Full text not available
312	Effect of moderate intensity exercise training on pulmonary functions in young normal and obese adults	No FEV1 or FVC data (mean ± SD)
313	Obesity and Spirometric Ventilatory Status Correlation in Adult Male Population of Amritsar. Number 31	Categories not clearly separated for analysis
314	Beneficial impact of weight loss on respiratory function in interstitial lung disease patients with obesity. Number 72	Categories not clearly separated for analysis
315	Gender differences in body composition, respiratory functions, life style among medical students. Number 75	Categories not clearly separated for analysis
316	Six-week inspiratory resistance training ameliorates endurance performance but does not affect obesity-related metabolic biomarkers in obese adults: A randomized controlled trial. Number 83	Categories not clearly separated for analysis
317	A pragmatic randomised controlled trial of tailored pulmonary rehabilitation in participants with difficult-to-control asthma and elevated body mass index. Number 94	Categories not clearly separated for analysis
318	Respiratory Effects of Treatment with a Glucagon-Like Peptide-1 Receptor Agonist in Patients Suffering from Obesity and Chronic Obstructive Pulmonary Disease. Number 112	Categories not clearly separated for analysis
319	Conflicting Role of Sarcopenia and Obesity in Male Patients with Chronic Obstructive Pulmonary Disease: Korean National Health and Nutrition Examination Survey	Categories not clearly separated for analysis
320	The effect of obesity on patients with mild chronic obstructive pulmonary disease: results from KNHANES 2010 to 2012	Categories not clearly separated for analysis
321	The effects of type 2 diabetes mellitus and its complications on physical and pulmonary functions: A case–control study	Categories not clearly separated for analysis
322	Challenging the obesity paradox: extreme obesity and COPD mortality in the SUMMIT trial	Categories not clearly separated for analysis
323	Effect of Body Mass Index on Lung Function in Chinese Patients with Chronic Obstructive Pulmonary Disease: A Multicenter Cross-Sectional Study	Categories not clearly separated for analysis
324	Impact of BMI on exacerbation and medical care expenses in subjects with mild to moderate airflow obstruction	Categories not clearly separated for analysis

325	Impact of Obesity on the Clinical Profile of a Population-Based Sample with Chronic Obstructive Pulmonary Disease	Categories not clearly separated for analysis
326	Obesity and COPD: Associated Symptoms, Health-related Quality of Life, and Medication Use	Categories not clearly separated for analysis
327	Obesity Is Associated With Increased Morbidity in Moderate to Severe COPD	Categories not clearly separated for analysis
328	Association between glycemic state and pulmonary function and effect of walking as a protective factor in subjects with diabetes mellitus	Categories not clearly separated for analysis
329	The effects of diet-induced weight loss on asthma control and quality of life in obese adults with asthma: a randomized controlled trial	Categories not clearly separated for analysis
330	Adiposity increases weight-bearing exercise-induced dyspnea despite favoring resting lung hyperinflation in COPD	Categories not clearly separated for analysis
331	Improvements of lung volumes and respiratory symptoms after weight loss through laparoscopic sleeve gastrectomy	Categories not clearly separated for analysis
332	Obesity affects pulmonary function in Japanese adult patients with asthma, but not those without asthma.	Categories not clearly separated for analysis
333	The relationship between general and abdominal obesity, nutrition and respiratory functions in adult asthmatics	Categories not clearly separated for analysis
334	Peripheral Airway Dysfunction in Obesity and Obese Asthma	Categories not clearly separated for analysis
