# Supplementary material for: The role of obesity and Type 2 diabetes in lung health: A systematic review (2024)
Source: PLoS One. 2026 Jan 23;21(1):e0340692. doi: 10.1371/journal.pone.0340692 (PMC12829954; doi:10.1371/journal.pone.0340692)
Supplement: S7 File — Quality assessment of cross-sectional studies (n = 74) using an adapted version of the Newcastle-Ottawa scale (See S3) (a). Studies were deemed as low, medium or high quality if they received 0–3, 4–5 or 6–7 stars, respectively. Quality assessment of cohort studies (n = 13) using the Newcastle-Ottawa scale for cohort studies (b). Studies were deemed as low, medium or high quality if they received 0–3, 4–7 or 8–10 stars, respectively. Quality assessment of case-control studies (n = 4) using the Newcastle-Ottawa scale for case-control studies (c). Studies were deemed as low, medium or high quality if they received 0–3, 4–6 or 7–9 stars, respectively. Risk of Bias for interventional studies (n = 2) using Cochrane Risk of Bias tool (RoB2) (d). Studies were rated as having either low, some concerns or high risk of bias. (DOCX) [file pone.0340692.s007.docx]

**S7: Quality assessment for cross-sectional studies (a), cohort studies (b), case-control studies (c), and Risk of Bias for interventional studies (d).**

1. Cross-sectional studies

| Title | Selection (3 Stars) | Comparability (2 Stars) | Outcome (2 Stars) | Number of Stars | Quality |
| --- | --- | --- | --- | --- | --- |
| Study of pulmonary function tests in diabetics with COPD or asthma | ٭٭٭ | ٭٭ | ٭ | 6 | High |
| Association of chronic obstructive pulmonary disease with type 2 diabetes mellitus | ٭٭ | ٭٭ | ٭ | 5 | Medium |
| A cross-sectional study connecting obesity and pulmonary function test among young adult in Northern India region | ٭٭٭ | ٭٭ | ٭٭ | 7 | High |
| A study on pulmonary function parameters in type 2 diabetes mellitus | ٭٭٭ | ٭٭ | ٭٭ | 7 | High |
| Alteration of pulmonary function in diabetic nephropathy | ٭٭٭ | ٭٭ | ٭٭ | 7 | High |
| Alveolar Gas Exchange and Pulmonary Functions in Patients with Type II Diabetes Mellitus | ٭٭٭ | ٭٭ | ٭٭ | 7 | High |
| Assessment of Pulmonary Functions in Obese Young Adults | ٭٭ | ٭ | ٭٭ | 5 | Medium |
| Assessment of pulmonary functions in type 2 diabetes mellitus: Its correlation with glycemic control and body mass index | ٭٭٭ | ٭٭ | ٭ | 6 | High |
| Association of body mass index with pulmonary function in overweight young adults | ٭ | ٭٭ | ٭٭ | 5 | Medium |
| Body Mass Index and Dynamic Lung Volumes in Office Workers | ٭٭٭ | ٭٭ | ٭٭ | 7 | High |
| Comparative study on differences in lung parameter between the obese and non obese collegiate sedentary students | ٭٭٭ | ٭٭ | ٭ | 6 | High |
| Duration of type 2 diabetes mellitus and pulmonary function tests: a correlative study | ٭٭ | ٭٭ | ٭ | 5 | Medium |
| Effect of Body Fat Distribution on Pulmonary Functions in Young Healthy Obese Students | ٭٭٭ | ٭٭ | ٭ | 6 | High |
| Effect of Body Mass Index on respiratory parameters: A cross-sectional analytical Study | ٭٭٭ | ٭٭ | ٭ | 6 | High |
| Effect of duration of diabetes on pulmonary functions in non-smoker type-2 diabetes mellitus | ٭٭٭ | ٭٭ | ٭٭ | 7 | High |
| Effect of Glycated Hemoglobin (HbA1c) and Duration of Disease on Lung Functions in Type 2 Diabetic Patients | ٭٭٭ | ٭٭ | ٭٭ | 7 | High |
| Effect of glycemic status on lung function tests in type 2 diabetes mellitus | ٭٭٭ | ٭٭ | ٭ | 6 | High |
| Effect of Obesity and Hypertension on Pulmonary Functions | ٭٭٭ | ٭٭ | ٭٭ | 7 | High |
| Effect of weight reduction on obese patients with COPD and bronchial asthma | ٭٭ | ٭٭ | ٭٭ | 6 | High |
| Effects of progressive increase in body weight on lung function in six groups of body mass index | ٭٭٭ | ٭ | ٭٭ | 6 | High |
| Impact Of Obesity on Pulmonary Functions Among Young Non-Smoker Healthy Female of Shah Alam, Malaysia | ٭٭ | ٭٭ | ٭ | 5 | Medium |
| Impact of overweight and obesity on ventilatory function among male medical students | ٭٭٭ | ٭٭ | ٭ | 6 | High |
| Lung Functions in Type 2 Diabetes Mellitus | ٭٭٭ | ٭٭ | ٭ | 6 | High |
| Metabolic Determinants of Impaired Pulmonary Function in Patients with Newly Diagnosed Type 2 Diabetes Mellitus | ٭٭٭ | ٭٭ | ٭٭ | 7 | High |
| Non‑linear association of anthropometric measurements and pulmonary function | ٭٭٭ | ٭٭ | ٭٭ | 7 | High |
| Obesity and Pulmonary Functions in Young Non Smoker Male of Shah Alam, Malaysia | ٭٭ | ٭٭ | ٭ | 5 | Medium |
| Pulmonary function changes in diabetic lung | ٭٭٭ | ٭٭ | ٭ | 6 | High |
| Pulmonary Function Tests and Their Associated Factors Among Type 2 Diabetic Patients at Jimma Medical Center, in 2019; Comparative Cross Sectional Study | ٭٭٭ | ٭٭ | ٭٭ | 7 | High |
| Pulmonary function tests in type 2 diabetes mellitus and their association with glycemic control and duration of the disease | ٭٭٭ | ٭ | ٭٭ | 6 | High |
| Pulmonary Function Tests in Type 2 Diabetics and Non-Diabetic People -A Comparative Study | ٭٭٭ | ٭٭ | ٭٭ | 7 | High |
| Reduction In Lung Functions in Type-2 Diabetes in Indian Population: Correlation With Glycemic Status | ٭٭٭ | ٭٭ | ٭٭ | 7 | High |
| Respiratory function in type II diabetes mellitus | ٭٭٭ | ٭٭ | ٭٭ | 7 | High |
| Restrictive pulmonary deficit is associated with inflammation in suboptimally controlled obese diabetics | ٭٭ | ٭ | ٭٭ | 5 | Medium |
| Study of lung function in patients of type 2 diabetes mellitus | ٭٭٭ | ٭٭ | ٭٭ | 7 | High |
| The Effect of Obesity on Pulmonary Function Testing Among the Jordanian Population | ٭٭٭ | ٭٭ | ٭٭ | 7 | High |
| The effects of body mass index on spirometry tests among adults in Xi’an, China | ٭٭ | ٭٭ | ٭٭ | 6 | High |
| The Relationship Between Anthropometric Measures, Blood Gases, and Lung Function in Morbidly Obese White Subjects | ٭٭ | ٭٭ | ٭٭ | 6 | High |
| The study of pulmonary functions tests and fat distribution in overweight and obese adult males | ٭٭٭ | ٭٭ | ٭ | 6 | High |
| Type II diabetes mellitus is associated with decreased measures of lung function in a clinical setting | ٭٭٭ | ٭٭ | ٭٭ | 7 | High |
| Determinants of exercise capacity in obese and non-obese COPD patients | ٭٭٭ | ٭٭ | ٭ | 6 | High |
| Diabetes Mellitus Type 2 in Hospitalized COPD Patients: Impact on Quality of Life and Lung Function | ٭٭٭ | ٭ | ٭ | 5 | Medium |
| Effect of obesity on respiratory mechanics during rest and exercise in COPD | ٭٭٭ | ٭ | ٭٭ | 6 | High |
| Grading the severity of obstruction in patients with Chronic Obstructive Pulmonary Disease and morbid obesity | ٭٭٭ | ٭ | ٭ | 5 | Medium |
| The impact of sex and BMI on the clinical course of COPD and bronchial asthma | ٭٭٭ | ٭ |  | 4 | Medium |
| Effects of BMI on static lung volumes in patients with airway obstruction | ٭٭٭ | ٭ | ٭٭ | 6 | High |
| Correlation of Pulmonary Function Tests with Anthropometry and Glycaemic Control in Type 2 Diabetes Mellitus: A Cross-sectional Study | ٭٭ | ٭٭ | ٭٭ | 6 | High |
| Effect of Type 2 Diabetes Mellitus on Pulmonary Function | ٭٭٭ | ٭٭ | ٭ | 6 | High |
| Gender Differences and Obesity Influence on Pulmonary Function Parameters | ٭٭٭ |  | ٭٭ | 5 | Medium |
| Mortality and Exacerbation Risk by Body Mass Index in Patients with COPD in TIOSPIR and UPLIFT | ٭٭٭ | ٭٭ | ٭٭ | 7 | High |
| Asthma diagnosis is not associated with obesity in a population of adults from Madrid | ٭٭٭ | ٭٭ | ٭٭ | 7 | High |
| Baseline of visceral fat area and decreased body weight correlate with improved pulmonary function after Roux-en-Y gastric bypass in Chinese obese patients with BMI 28-35 kg/m2 and type 2 diabetes: a 6-month follow-up | ٭٭ | ٭ | ٭٭ | 5 | Medium |
| Effect of obesity on asthma phenotype is dependent upon asthma severity | ٭٭٭ | ٭٭ | ٭٭ | 7 | High |
| Influence of body mass indexes on response to treatment in acute asthma | ٭٭٭ | ٭٭ | ٭٭ | 7 | High |
| Lung age in women with morbid obesity | ٭٭٭ | ٭٭ | ٭٭ | 7 | High |
| Observational study of the effect of obesity on lung volumes | ٭٭٭ | ٭٭ | ٭٭ | 7 | High |
| Reduced pulmonary functions and respiratory muscle strength in Type 2 diabetes mellitus and its association with glycemic control | ٭٭٭ | ٭ | ٭٭ | 6 | High |
| Relationship between pulmonary function and albuminuria in type 2 diabetic patients with preserved renal function | ٭٭٭ | ٭٭ | ٭٭ | 7 | High |
| Spirometric values in elderly asthmatic patients are not influenced by obesity | ٭٭٭ | ٭ | ٭٭ | 6 | High |
| Total and Compartmental Chest Wall Volumes, Lung Function, and Respiratory Muscle Strength in Individuals with Abdominal Obesity: Effects of Body Positions | ٭٭ | ٭٭ | ٭٭ | 6 | High |
| Association body mass index and spirometric lung function in chronic obstructive pulmonary disease (COPD) patients attending RIMS Hospital, Manipur | ٭٭٭ | ٭ | ٭ | 5 | Medium |
| The impact of abdominal adiposity measured by sonography on the pulmonary function of pre-menopausal females | ٭٭٭ | ٭٭ | ٭٭ | 7 | High |
| Status of Pulmonary function in Indian young overweight male individuals | ٭٭٭ | ٭٭ | ٭٭ | 7 | High |
| A comparative study of FVC, FEV1, FEV1/FVC ratio before and after cycling in young obese and non-obese women | ٭٭٭ | ٭٭ | ٭ | 6 | High |
| A new approach for the detection of obesity-related airway obstruction in lung-healthy individuals | ٭٭ | ٭٭ | ٭٭ | 6 | High |
| A Study of Correlation of Pulmonary Function Tests and Body Mass Index in the MBBS Students and Health Care Workers of Bhagwan Mahavir Institute of Medical Sciences, Pawapuri | ٭٭٭ | ٭ | ٭ | 5 | Medium |
| Asthma phenotype: Clinical, physiological, and biochemical profiles of North Indian patients | ٭٭٭ | ٭٭ | ٭٭ | 7 | High |
| Comparison of FEV1/FVC in Type-2 Diabetes Mellitus Patients and Healthy Individuals | ٭٭٭ | ٭٭ | ٭٭ | 7 | High |
| Disorders of Pulmonary Function in Type 2 Diabetes Mellitus Patients With Different Types of Oral Hypoglycemic Medications: Metformin, Metformin Plus Thiazolidinedione and Metformin plus Sulfonylurea | ٭٭٭ | ٭٭ | ٭٭ | 7 | High |
| Down-regulated surfactant protein B in obese asthmatics | ٭٭٭ | ٭ |  | 4 | Medium |
| Dynamic diffusion lung capacity of carbon monoxide (DLCO) as a predictor of pulmonary microangiopathy and its association with extra pulmonary microangiopathy in patients with type II diabetes mellitus | ٭٭٭ | ٭٭ | ٭٭ | 7 | High |
| Effect of age, gender, and body mass index on peak expiratory flow rate and other pulmonary function tests in healthy individuals in the age group 18-60 years | ٭٭٭ | ٭ | ٭٭ | 6 | High |
| Increased airway resistance can be related to the decrease in the functional capacity in obese women | ٭٭ | ٭٭ | ٭٭ | 6 | High |
| Physical activity levels in asthma: relationship with disease severity, body mass index and novel accelerometer-derived metrics | ٭٭٭ | ٭٭ | ٭ | 6 | High |
| Study of Pulmonary Function Tests in Diabetic Nephropathy | ٭٭٭ | ٭٭ | ٭٭ | 7 | High |

1. Cohort studies

| Title | Selection (4 Stars) | Comparability (2 Stars) | Outcome (4 Stars) | Number of Stars | Quality |
| --- | --- | --- | --- | --- | --- |
| Obesity and functioning among individuals with chronic obstructive pulmonary disease (COPD) | ٭٭٭٭ | ٭٭ | ٭٭٭٭ | 10 | High |
| Reduced lung function is independently associated with increased risk of type 2 diabetes in Korean men | ٭٭٭٭ | ٭ | ٭٭٭٭ | 9 | High |
| A prospective study on physical performance of Chinese chronic obstructive pulmonary disease males with type 2 diabetes | ٭٭٭ | ٭٭ | ٭٭ | 7 | Medium |
| Impact of diabetes mellitus on the risk of severe exacerbation in patients with chronic obstructive pulmonary disease | ٭٭٭ | ٭ | ٭٭٭ | 7 | Medium |
| Obesity in chronic obstructive pulmonary disease: Is fatter really better? | ٭٭٭٭ | ٭٭ | ٭٭٭٭ | 10 | High |
| Decline of the lung function and quality of glycemic control in type 2 diabetes mellitus | ٭٭٭٭ | ٭٭ | ٭٭٭٭ | 10 | High |
| Airway hyperresponsiveness is negatively associated with obesity or overweight status in patients with asthma | ٭٭٭٭ | ٭٭ | ٭٭٭٭ | 10 | High |
| Functional lung rejuvenation in obese patients after bariatric surgery | ٭٭٭٭ | ٭٭ | ٭٭٭٭ | 10 | High |
| Overweight is Associated with Airflow Obstruction and Poor Disease Control but Not with Exhaled Nitric Oxide Change in an Asthmatic Population | ٭٭٭٭ | ٭٭ | ٭٭٭٭ | 10 | High |
| Surgically induced weight loss, including reduction in waist circumference, is associated with improved pulmonary function in obese patients | ٭٭٭٭ | ٭ | ٭٭٭٭ | 9 | High |
| Obesity is a determinant of asthma control independent of inflammation and lung mechanics | ٭٭٭٭ | ٭٭ | ٭٭٭ | 9 | High |
| Body mass index increase: a risk factor for forced expiratory volume in 1 s decline for overweight and obese adults with asthma | ٭٭٭٭ | ٭٭ | ٭٭٭٭ | 10 | High |
| Obesity in women with asthma: Baseline disadvantage plus greater small-airway responsiveness | ٭٭٭٭ | ٭٭ | ٭٭٭ | 9 | High |

1. Case-control studies

| Title | Selection (4 Stars) | Comparability (2 Stars) | Outcome (3 Stars) | Number of Stars | Quality |
| --- | --- | --- | --- | --- | --- |
| Pulmonary function tests in type 2 diabetes mellitus and their association with glycemic status and insulin resistance | ٭٭٭ | ٭٭ | ٭ | 6 | Medium |
| Serum Surfactant Protein D as a Biomarker for Measuring Lung Involvement in Obese Patients With Type 2 Diabetes | ٭٭٭ | ٭٭ | ٭٭ | 7 | High |
| A Study on Pulmonary Function Tests In Type 2 Diabetes Mellitus Patients- A Case Control Study From South India | ٭٭٭٭ | ٭٭ | ٭ | 7 | High |
| Allergic and non-allergic asthma phenotypes and exposure to air pollution | ٭٭٭٭ | ٭٭ | ٭٭٭ | 9 | High |

1. Interventional Studies

| Title | Randomisation Process | Deviations from the Intended Interventions | Missing Outcome Data | Measurement of the Outcome | Selection of the Reported Result | Overall Risk of Bias |
| --- | --- | --- | --- | --- | --- | --- |
| Effect of Glucose Improvement on Spirometric Maneuvers in Patients With Type 2 Diabetes: The Sweet Breath Study | Some Concerns | Low | Low | Low | Low | Some Concerns |
| Liraglutide Improves Forced Vital Capacity in Individuals With Type 2 Diabetes: Data From the Randomized Crossover LIRALUNG Study | Low | Low | Low | Low | Low | Low |

Quality assessment of cross-sectional studies (n=74) using an adapted version of the Newcastle-Ottawa scale (See S3) (a). Studies were deemed as low, medium or high quality if they received 0-3, 4-5 or 6-7 stars, respectively. Quality assessment of cohort studies (n=13) using the Newcastle-Ottawa scale for cohort studies (b). Studies were deemed as low, medium or high quality if they received 0-3, 4-7 or 8-10 stars, respectively. Quality assessment of case-control studies (n=4) using the Newcastle-Ottawa scale for case-control studies (c). Studies were deemed as low, medium or high quality if they received 0-3, 4-6 or 7-9 stars, respectively. Risk of Bias for interventional studies (n=2) using Cochrane Risk of Bias tool (RoB2) (d). Studies were rated as having either low, some concerns or high risk of bias.
