# Supplementary material for: The role of obesity and Type 2 diabetes in lung health: A systematic review (2024)
Source: PLoS One. 2026 Jan 23;21(1):e0340692. doi: 10.1371/journal.pone.0340692 (PMC12829954; doi:10.1371/journal.pone.0340692)
Supplement: S8 File — Statistical significance between all study categories for age, BMI, FEV1% of predicted, FVC% of predicted and FEV1/FVC (L/L%) ratio is presented and separated by BMI status. Data presented as mean ± standard deviation. BMI body mass index; FEV1 forced expiratory volume in one second; FVC forced vital capacity; COPD chronic obstructive pulmonary disease; T2D Type 2 diabetes. * P ≤ 0.05, ** P ≤ 0.01, *** P ≤ 0.001, **** P ≤ 0.0001. Lean BMI 18.5–24.9 kg/m2, overweight BMI 25–29.9 kg/m2, obese BMI ≥ 30 kg/m2. (DOCX) [file pone.0340692.s008.docx]

**S8: BMI, age, FEV1, FVC and FEV1/FVC ratio separated by BMI category.**


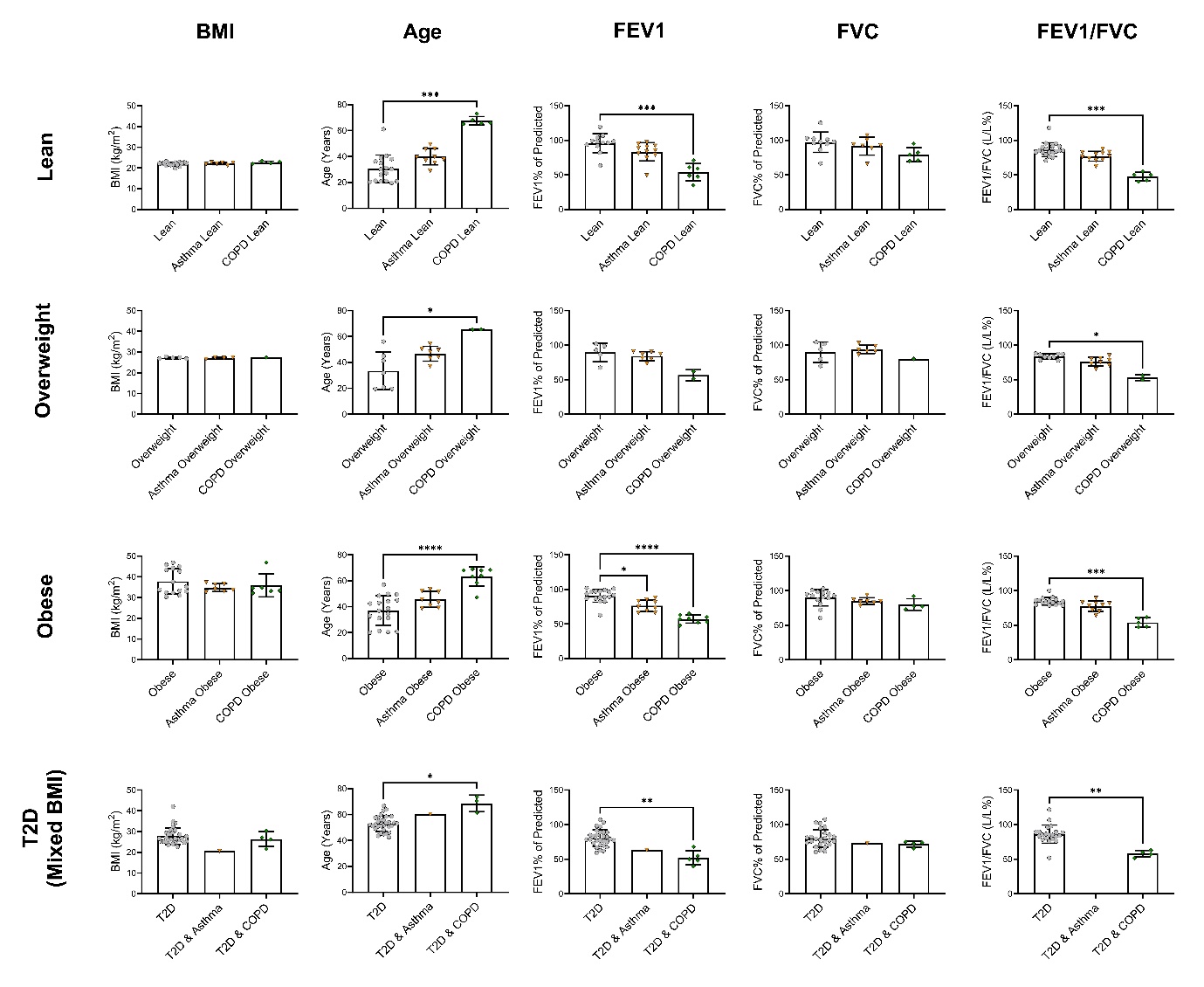


Statistical significance between all study categories for age, BMI, FEV1% of predicted, FVC% of predicted and FEV1/FVC (L/L%) ratio is presented and separated by BMI status. Data presented as mean ± standard deviation. *BMI* body mass index; *FEV1* forced expiratory volume in one second; *FVC* forced vital capacity; *COPD* chronic obstructive pulmonary disease; *T2D* Type 2 diabetes. * *P* ≤0.05, ** *P* ≤0.01, *** *P* ≤0.001, **** *P* ≤0.0001. Lean BMI 18.5-24.9 kg/m^2^, overweight BMI 25-29.9 kg/m^2^, obese BMI≥30 kg/m^2^.
