# Supplementary material for: The role of obesity and Type 2 diabetes in lung health: A systematic review (2024)
Source: PLoS One. 2026 Jan 23;21(1):e0340692. doi: 10.1371/journal.pone.0340692 (PMC12829954; doi:10.1371/journal.pone.0340692)
Supplement: S11 File — (DOCX) [file pone.0340692.s011.docx]

**S11: Quality assessment and risk of bias results.**

(a). Cross-Sectional Studies

|  | **Selection (3 Stars)** | | | **Comparability (2 Stars)** | |  |
| --- | --- | --- | --- | --- | --- | --- |
|  | Representativeness of the sample | Selection of the control cohort | Ascertainment of the exposure | Study Controls for … | Study Controls Additional Factors | **Outcome (2 Stars)** |
| Study of pulmonary function tests in diabetics with COPD or asthma | * | * | * | * | * | * |
| Association of chronic obstructive pulmonary disease with type 2 diabetes mellitus | * | - | * | * | * | * |
| A cross-sectional study connecting obesity and pulmonary function test among young adult in Northern India region | * | * | * | * | * | ** |
| A study on pulmonary function parameters in type 2 diabetes mellitus | * | * | * | * | * | ** |
| Alteration of pulmonary function in diabetic nephropathy | * | * | * | * | * | ** |
| Alveolar Gas Exchange and Pulmonary Functions in Patients with Type II Diabetes Mellitus | * | * | * | * | * | ** |
| Assessment of Pulmonary Functions in Obese Young Adults | - | * | * | * | - | ** |
| Assessment of pulmonary functions in type 2 diabetes mellitus: Its correlation with glycemic control and body mass index | * | * | * | * | * | * |
| Association of body mass index with pulmonary function in overweight young adults | - | - | * | * | * | ** |
| Body Mass Index and Dynamic Lung Volumes in Office Workers | * | * | * | * | * | ** |
| Comparative study on differences in lung parameter between the obese and non obese collegiate sedentary students | * | * | * | * | * | * |
| Duration of type 2 diabetes mellitus and pulmonary function tests: a correlative study | * | - | * | * | * | * |
| Effect of Body Fat Distribution on Pulmonary Functions in Young Healthy Obese Students | * | * | * | * | * | * |
| Effect of Body Mass Index on respiratory parameters: A cross-sectional analytical Study | * | * | * | * | * | * |
| Effect of duration of diabetes on pulmonary functions in non-smoker type-2 diabetes mellitus | * | * | * | * | * | ** |
| Effect of Glycated Hemoglobin (HbA1c) and Duration of Disease on Lung Functions in Type 2 Diabetic Patients | * | * | * | * | * | ** |
| Effect of glycemic status on lung function tests in type 2 diabetes mellitus | * | * | * | * | * | * |
| Effect of Obesity and Hypertension on Pulmonary Functions | * | * | * | * | * | ** |
| Effect of weight reduction on obese patients with COPD and bronchial asthma | * | - | * | * | * | ** |
| Effects of progressive increase in body weight on lung function in six groups of body mass index | * | * | * | * | - | ** |
| Impact Of Obesity on Pulmonary Functions Among Young Non-Smoker Healthy Female of Shah Alam, Malaysia | * | * | - | * | * | * |
| Impact of overweight and obesity on ventilatory function among male medical students | * | * | * | * | * | * |
| Lung Functions in Type 2 Diabetes Mellitus | * | * | * | * | * | * |
| Metabolic Determinants of Impaired Pulmonary Function in Patients with Newly Diagnosed Type 2 Diabetes Mellitus | * | * | * | * | * | ** |
| Non‑linear association of anthropometric measurements and pulmonary function | * | * | * | * | * | ** |
| Obesity and Pulmonary Functions in Young Non Smoker Male of Shah Alam, Malaysia | * | * | - | * | * | * |
| Pulmonary function changes in diabetic lung | * | * | * | * | * | * |
| Pulmonary Function Tests and Their Associated Factors Among Type 2 Diabetic Patients at Jimma Medical Center, in 2019; Comparative Cross Sectional Study | * | * | * | * | * | ** |
| Pulmonary function tests in type 2 diabetes mellitus and their association with glycemic control and duration of the disease | * | * | * | * | - | ** |
| Pulmonary Function Tests in Type 2 Diabetics and Non-Diabetic People -A Comparative Study | * | * | * | * | * | ** |
| Reduction In Lung Functions in Type-2 Diabetes in Indian Population: Correlation With Glycemic Status | * | * | * | * | * | ** |
| Respiratory function in type II diabetes mellitus | * | * | * | * | * | ** |
| Restrictive pulmonary deficit is associated with inflammation in suboptimally controlled obese diabetics | * | - | * | * | - | ** |
| Study of lung function in patients of type 2 diabetes mellitus | * | * | * | * | * | ** |
| The Effect of Obesity on Pulmonary Function Testing Among the Jordanian Population | * | * | * | * | * | ** |
| The effects of body mass index on spirometry tests among adults in Xi’an, China | * | * | - | * | * | ** |
| The Relationship Between Anthropometric Measures, Blood Gases, and Lung Function in Morbidly Obese White Subjects | * | - | * | * | * | ** |
| The study of pulmonary functions tests and fat distribution in overweight and obese adult males | * | * | * | * | * | * |
| Type II diabetes mellitus is associated with decreased measures of lung function in a clinical setting | * | * | * | * | * | ** |
| Determinants of exercise capacity in obese and non-obese COPD patients | * | * | * | * | * | * |
| Diabetes Mellitus Type 2 in Hospitalized COPD Patients: Impact on Quality of Life and Lung Function | * | * | * | * | - | * |
| Effect of obesity on respiratory mechanics during rest and exercise in COPD | * | * | * | * | - | ** |
| Grading the severity of obstruction in patients with Chronic Obstructive Pulmonary Disease and morbid obesity | * | * | * | * | - | * |
| The impact of sex and BMI on the clinical course of COPD and bronchial asthma | * | * | * | * | - | - |
| Effects of BMI on static lung volumes in patients with airway obstruction | * | * | * | * | - | ** |
| Correlation of Pulmonary Function Tests with Anthropometry and Glycaemic Control in Type 2 Diabetes Mellitus: A Cross-sectional Study | * | - | * | * | * | ** |
| Effect of Type 2 Diabetes Mellitus on Pulmonary Function | * | * | * | * | * | * |
| Gender Differences and Obesity Influence on Pulmonary Function Parameters | * | * | * | - | - | ** |
| Mortality and Exacerbation Risk by Body Mass Index in Patients with COPD in TIOSPIR and UPLIFT | * | * | * | * | * | ** |
| Asthma diagnosis is not associated with obesity in a population of adults from Madrid | * | * | * | * | * | ** |
| Baseline of visceral fat area and decreased body weight correlate with improved pulmonary function after Roux-en-Y gastric bypass in Chinese obese patients with BMI 28-35 kg/m2 and type 2 diabetes: a 6-month follow-up | * | - | * | * | - | ** |
| Effect of obesity on asthma phenotype is dependent upon asthma severity | * | * | * | * | * | ** |
| Influence of body mass indexes on response to treatment in acute asthma | * | * | * | * | * | ** |
| Lung age in women with morbid obesity | * | * | * | * | * | ** |
| Observational study of the effect of obesity on lung volumes | * | * | * | * | * | ** |
| Reduced pulmonary functions and respiratory muscle strength in Type 2 diabetes mellitus and its association with glycemic control | * | * | * | * | - | ** |
| Relationship between pulmonary function and albuminuria in type 2 diabetic patients with preserved renal function | * | * | * | * | * | ** |
| Spirometric values in elderly asthmatic patients are not influenced by obesity | * | * | * | * | - | ** |
| Total and Compartmental Chest Wall Volumes, Lung Function, and Respiratory Muscle Strength in Individuals with Abdominal Obesity: Effects of Body Positions | * | * | - | * | * | ** |
| Association body mass index and spirometric lung function in chronic obstructive pulmonary disease (COPD) patients attending RIMS Hospital, Manipur | * | * | * | * | - | * |
| The impact of abdominal adiposity measured by sonography on the pulmonary function of pre-menopausal females | * | * | * | * | * | ** |
| Status of Pulmonary function in Indian young overweight male individuals | * | * | * | * | * | ** |
| A comparative study of FVC, FEV1, FEV1/FVC ratio before and after cycling in young obese and non-obese women | * | * | * | * | * | * |
| A new approach for the detection of obesity-related airway obstruction in lung-healthy individuals | * | - | * | * | * | ** |
| A Study of Correlation of Pulmonary Function Tests and Body Mass Index in the MBBS Students and Health Care Workers of Bhagwan Mahavir Institute of Medical Sciences, Pawapuri | * | * | * | * | - | * |
| Asthma phenotype: Clinical, physiological, and biochemical profiles of North Indian patients | * | * | * | * | * | ** |
| Comparison of FEV1/FVC in Type-2 Diabetes Mellitus Patients and Healthy Individuals | * | * | * | * | * | ** |
| Disorders of Pulmonary Function in Type 2 Diabetes Mellitus Patients With Different Types of Oral Hypoglycemic Medications: Metformin, Metformin Plus Thiazolidinedione and Metformin plus Sulfonylurea | * | * | * | * | * | ** |
| Down-regulated surfactant protein B in obese asthmatics | * | * | * | * | - | - |
| Dynamic diffusion lung capacity of carbon monoxide (DLCO) as a predictor of pulmonary microangiopathy and its association with extra pulmonary microangiopathy in patients with type II diabetes mellitus | * | * | * | * | * | ** |
| Effect of age, gender, and body mass index on peak expiratory flow rate and other pulmonary function tests in healthy individuals in the age group 18-60 years | * | * | * | * | - | ** |
| Increased airway resistance can be related to the decrease in the functional capacity in obese women | * | - | * | * | * | ** |
| Physical activity levels in asthma: relationship with disease severity, body mass index and novel accelerometer-derived metrics | * | * | * | * | * | * |
| Study of Pulmonary Function Tests in Diabetic Nephropathy | * | * | * | * | * | ** |

Quality assessment of cross-sectional studies (n=74) using an adapted version of the Newcastle-Ottawa scale (Supporting Information, S4). Studies were deemed as low, medium, or high quality if they received 0-3, 4-5 or 6-7 stars, respectively, for cross-sectional studies.

(b). Cohort Studies

|  | **Selection (4 Stars)** | | | | **Comparability (2 Stars)** | | **Outcome (4 Stars)** | | |
| --- | --- | --- | --- | --- | --- | --- | --- | --- | --- |
|  | Representativeness of the exposed cohort | Selection of the non-exposed cohort | Ascertainment of exposure | Demonstration that outcome of interest was not present at start of study | Study Controls for … | Study Controls Additional Factors | Assessment of outcome | Was follow-up long enough for outcomes to occur | Adequacy of follow up of cohorts |
| Obesity and functioning among individuals with chronic obstructive pulmonary disease (COPD) | * | * | * | * | * | * | ** | * | * |
| Reduced lung function is independently associated with increased risk of type 2 diabetes in Korean men | * | * | * | * | * | - | ** | * | * |
| A prospective study on physical performance of Chinese chronic obstructive pulmonary disease males with type 2 diabetes | * | - | * | * | * | * | ** | - | - |
| Impact of diabetes mellitus on the risk of severe exacerbation in patients with chronic obstructive pulmonary disease | * | * | - | * | * | - | ** | * | - |
| Obesity in chronic obstructive pulmonary disease: Is fatter really better? | * | * | * | * | * | * | ** | * | * |
| Decline of the lung function and quality of glycemic control in type 2 diabetes mellitus | * | * | * | * | * | * | ** | * | * |
| Airway hyperresponsiveness is negatively associated with obesity or overweight status in patients with asthma | * | * | * | * | * | * | ** | * | * |
| Functional lung rejuvenation in obese patients after bariatric surgery | * | * | * | * | * | * | ** | * | * |
| Overweight is Associated with Airflow Obstruction and Poor Disease Control but Not with Exhaled Nitric Oxide Change in an Asthmatic Population | * | * | * | * | * | * | ** | * | * |
| Surgically induced weight loss, including reduction in waist circumference, is associated with improved pulmonary function in obese patients | * | * | * | * | * | - | ** | * | * |
| Obesity is a determinant of asthma control independent of inflammation and lung mechanics | * | * | * | * | * | * | ** | * | - |
| Body mass index increase: a risk factor for forced expiratory volume in 1 s decline for overweight and obese adults with asthma | * | * | * | * | * | * | ** | * | * |
| Obesity in women with asthma: Baseline disadvantage plus greater small-airway responsiveness | * | * | * | * | * | * | * | * | * |

Quality assessment of cohort studies (n=13) using an adapted version of the Newcastle-Ottawa scale (Supporting Information, S5). The cohort studies were deemed as low, medium, or high quality if they received 0-3, 4-7 or 8-10 stars, respectively.

(c). Case-Control Studies

|  | **Selection (4 Stars)** | | | | **Comparability (2 Stars)** | | **Exposure (3 Stars)** | | |
| --- | --- | --- | --- | --- | --- | --- | --- | --- | --- |
|  | Is the case definition adequate? | Representativeness of the cases | Selection of Controls | Definition of Controls | Study Controls for … | Study Controls Additional Factors | Ascertainment of exposure | Same method of ascertainment for cases and controls | Non-Response rate |
| Pulmonary function tests in type 2 diabetes mellitus and their association with glycemic status and insulin resistance | * | * | - | * | * | * | - | * | - |
| Serum Surfactant Protein D as a Biomarker for Measuring Lung Involvement in Obese Patients With Type 2 Diabetes | * | * | - | * | * | * | * | * | - |
| A Study on Pulmonary Function Tests In Type 2 Diabetes Mellitus Patients- A Case Control Study From South India | * | * | * | * | * | * | - | - | * |
| Allergic and non-allergic asthma phenotypes and exposure to air pollution | * | * | * | * | * | * | * | * | * |

Quality assessment of case-control studies (n=4) using the Newcastle-Ottawa scale for case-control studies. The case-control studies were deemed as low, medium, or high quality if they received 0-3, 4-6 or 7-9 stars, respectively.

(d). Interventional Studies

|  |  | **Effect of Glucose Improvement on Spirometric Maneuvers in Patients With Type 2 Diabetes: The Sweet Breath Study** | **Liraglutide Improves Forced Vital Capacity in Individuals With Type 2 Diabetes: Data From the Randomized Crossover LIRALUNG Study** |
| --- | --- | --- | --- |
| **Randomisation Process** | 1.1 Was the allocation sequence random? | NA | Y |
|  | 1.2 Was the allocation sequence concealed until participants were enrolled and assigned to interventions? | NA | Y |
|  | 1.3 Did baseline differences between intervention groups suggest a problem with the randomization process? | NA | N |
|  | Risk-of-bias judgement | Some Concerns | Low |
| **Deviations from the Intended Interventions** (effect of adhering to intervention) | 2.1. Were participants aware of their assigned intervention during the trial? | Y | N |
|  | 2.2. Were carers and people delivering the interventions aware of participants' assigned intervention during the trial? | Y | N |
|  | 2.3. [If applicable:] If Y/PY/NI to 2.1 or 2.2: Were important non-protocol interventions balanced across intervention groups? | Y | NA |
|  | 2.4. [If applicable:] Were there failures in implementing the intervention that could have affected the outcome? | PN | PN |
|  | 2.5. [If applicable:] Was there non-adherence to the assigned intervention regimen that could have affected participants’ outcomes? | PN | PN |
|  | 2.6. If N/PN/NI to 2.3, or Y/PY/NI to 2.4 or 2.5: Was an appropriate analysis used to estimate the effect of adhering to the intervention? | NA | NA |
|  | Risk-of-bias judgement | Low | Low |
| **Missing Outcome Data** | 3.1 Were data for this outcome available for all, or nearly all, participants randomized? | Y | PY |
|  | 3.2 If N/PN/NI to 3.1: Is there evidence that the result was not biased by missing outcome data? | NA | NA |
|  | 3.3 If N/PN to 3.2: Could missingness in the outcome depend on its true value? | NA | NA |
|  | 3.4 If Y/PY/NI to 3.3: Is it likely that missingness in the outcome depended on its true value? | NA | NA |
|  | Risk-of-bias judgement | Low | Low |
| **Measurement of the Outcome** | 4.1 Was the method of measuring the outcome inappropriate? | PN | PN |
|  | 4.2 Could measurement or ascertainment of the outcome have differed between intervention groups? | N | N |
|  | 4.3 If N/PN/NI to 4.1 and 4.2: Were outcome assessors aware of the intervention received by study participants? | Y | N |
|  | 4.4 If Y/PY/NI to 4.3: Could assessment of the outcome have been influenced by knowledge of intervention received? | PN | NA |
|  | 4.5 If Y/PY/NI to 4.4: Is it likely that assessment of the outcome was influenced by knowledge of intervention received? | PN | NA |
|  | Risk-of-bias judgement | Low | Low |
| **Selection of the Reported Result** | 5.1 Were the data that produced this result analysed in accordance with a pre-specified analysis plan that was finalized before unblinded outcome data were available for analysis? | PY | Y |
|  | 5.2. Is the numerical result being assessed likely to have been selected, on the basis of the results, from multiple eligible outcome measurements (e.g. scales, definitions, time points) within the outcome domain? | N | PN |
|  | 5.3 Is the numerical result being assessed likely to have been selected, on the basis of the results, from multiple eligible analyses of the data? | PN | PN |
|  | Risk-of-bias judgement | Low | Low |

Risk of Bias was used for interventional studies (n=2) using the Cochrane risk-of-bias tool for randomised trials (RoB 2) methodology. Studies were rated as having either low, some concerns or high risk of bias. Y = Yes, N = No, PY = Probably Yes, PN= Probably No, NA = Not Applicable.
