# Supplementary material for: The role of obesity and Type 2 diabetes in lung health: A systematic review (2024)
Source: PLoS One. 2026 Jan 23;21(1):e0340692. doi: 10.1371/journal.pone.0340692 (PMC12829954; doi:10.1371/journal.pone.0340692)
Supplement: S12 File — Multiple linear regression of FEV1/FVC. Model 1 is Type 2 diabetes alone, model 2 is with the addition of BMI, model 3 with the addition of geographically region and model 4 with the addition of age. Analysis was conducted on never smokers without an established lung disease. None of the models were normally distributed. SE standard error; VIF variance inflation factor; CI confidence interval. (DOCX) [file pone.0340692.s012.docx]

**S12:** **Multiple linear regression of FEV1/FVC.**

| Model | Predictor | Estimate (β) | SE | 95% CI | VIF | P value | Goodness of Fit (R^2^) |
| --- | --- | --- | --- | --- | --- | --- | --- |
| 1 | Intercept | 87.03 | 2.008 | [82.98, 91.07] |  | <0.0001 |  |
|  | T2DM [Yes] | -0.6813 | 3.338 | [-7.41, 6.04] | 1.000 | 0.8392 | 0.0009 |
|  |  |  |  |  |  |  |  |
| 2 | Intercept | 83.47 | 7.694 | [67.78, 99.16] |  | **<0.0001** |  |
|  | BMI | 0.05309 | 0.27 | [-0.50, 0.60] | 1.029 | 0.8453 |  |
|  | T2DM [Yes] | -2.365 | 3.063 | [-8.61, 3.88] | 1.029 | 0.4458 | 0.0223 |
|  |  |  |  |  |  |  |  |
| 3 | Intercept | 76.98 | 12.44 | [51.46, 102.5] |  | <0.0001 |  |
|  | Region [Asia] | 5.243 | 8.895 | [-13.01, 23.49] | 8.342 | 0.5605 |  |
|  | Region [Middle East] | 14.5 | 9.337 | [-4.659, 33.65] | 5.268 | 0.1321 |  |
|  | Region [Africa] | 3.068 | 11.91 | [-21.37, 27.50] | 1.950 | 0.7987 |  |
|  | Region [Latin America] | 4.504 | 9.943 | [-15.90, 24.91] | 4.945 | 0.6542 |  |
|  | BMI | 0.07022 | 0.278 | [-0.50, 0.64] | 1.162 | 0.8025 |  |
|  | T2DM [Yes] | -3.008 | 3.273 | [-9.72, 3.71] | 1.250 | 0.3663 | 0.1991 |
|  |  |  |  |  |  |  |  |
| 4 | Intercept | 88.42 | 14.58 | [58.39, 118.5] |  | <0.0001 |  |
|  | Region [Asia] | 1.715 | 9.077 | [-16.98, 20.41] | 8.753 | 0.8516 |  |
|  | Region [Middle East] | 11.45 | 9.447 | [-8.010, 30.90] | 5.485 | 0.237 |  |
|  | Region [Africa] | -0.9132 | 12.04 | [-25.72, 23.89] | 2.038 | 0.9402 |  |
|  | Region [Latin America] | 2.726 | 9.913 | [-17.69, 23.14] | 5.004 | 0.7856 |  |
|  | BMI | 0.1511 | 0.285 | [-0.44, 0.74] | 1.192 | 0.6012 |  |
|  | Age | -0.3635 | 0.227 | [-0.83, 0.10] | 4.869 | 0.1217 |  |
|  | T2DM [Yes] | 6.365 | 6.651 | [-7.33, 20.06] | 5.166 | 0.3477 | 0.2742 |

Multiple linear regression of FEV1/FVC. Model 1 is Type 2 diabetes alone, model 2 is with the addition of BMI, model 3 with the addition of geographically region and model 4 with the addition of age. Analysis was conducted on never smokers without an established lung disease. None of the models were normally distributed. SE standard error; VIF variance inflation factor; CI confidence interval.
