# Supplementary material for: The role of obesity and Type 2 diabetes in lung health: A systematic review (2024)
Source: PLoS One. 2026 Jan 23;21(1):e0340692. doi: 10.1371/journal.pone.0340692 (PMC12829954; doi:10.1371/journal.pone.0340692)
Supplement: Attachment — Submitted filename: PLOS ONE Correction List V2(1).docx [file pone.0340692.s014.docx]

**PLOS ONE Correction List**

We are grateful to the edtorial team and both reviewers for their detailed assessment of our manucript. We have implemented the suggested changes where possible and trust that the manuscript has been improved as a result. All data has been re-analysed and we have strictly excluded any papers where co-morbidities may have confounded the original data. This has reduced the overall number of papers in the systematic review from 109 to 93. Despite this, our original findings stand. Please find a point-by-point response below with our answers to queries provided in blue text.

**Journal Required Corrections:**

1. **Re-check document style requirements**

Please ensure that your manuscript meets PLOS ONE's style requirements, including those for file naming.

**Response:**

- Included page numbers and line numbers
- Manuscript is double-spaced
- Funding not included in the main manuscript
- Title: meets character limit (250 characters)
- Abstract: does not exceed 300 words (281)
- CRediT taxonomy: all author contributions have been updated to CRediT
- Headings: the methods section has been limited to 3 heading levels and the results section to 3.
- Regression analysis: Sentence added “The variance inflation factor (VIF) is presented for examination of multicollinearity.” As per the author guidelines, all regression analysis needs to be included in the supplementary files. Therefore, the FEV1/FVC regression analysis has been added as Supporting Information, S12.
- Ending section in this order: Acknowledgments, References, Supporting Information Captions
- Tables: placed immediately after the first paragraph in which they are cited and not uploaded in separate files
- Supporting Information naming: All supporting information documents have been labelled with ‘S’ and a number. Supporting information is not necessarily cited in order as per the author guidelines “If you cite supporting information in the text, citations do not need to be in numerical order.”
- Figures: abbreviated to ‘Fig’ in the figure captions and on the names of the files. Figures are not included in main manuscript.
- PRISMA checklist: included as supporting information S9 and S10. Also added the sentence “The PRISMA checklists are available in Supporting Information S9 and S10.”
- Tracked changes manuscript labelled: Revised Manuscript with Track Changes
- Unmarked version of revised paper without tracked changes labelled: Manuscript

2. As required by our policy on Data Availability, please ensure your manuscript or supplementary information includes the following:
1. **Add list of excluded studies.**

A numbered table of all studies identified in the literature search, including those that were excluded from the analyses. For every excluded study, the table should list the reason(s) for exclusion.

**Response:** A full list of all articles removed after the second screening (i.e. 334) and the included 93 articles are available in Supporting Information S6.

2. **Confirm if any are unpublished**

If any of the included studies are unpublished, include a link (URL) to the primary source or detailed information about how the content can be accessed.

**Response:** No included article is unpublished

3. **Table Containing All Data**

A table of all data extracted from the primary research sources for the systematic review and/or meta-analysis. This information can be included in the main text, supplementary information, or relevant data repository. The table must include the following information for each study:

a. Name of data extractors and date of data extraction

**Response:** Name of data extractors and date of data extraction has been added to ‘Supporting Information, S6’

b. Confirmation that the study was eligible to be included in the review.

**Response:** All articles in the ‘keep’ tab of the excel spreadsheet ‘Supporting Information, S6’ are confirmed to be eligible to be included in the review. Those that were not eligible are contained in the ‘Not Keep’ tab.

c. **Include all extracted data**

All data extracted from each study for the reported systematic review and/or meta-analysis that would be needed to replicate your analyses.

**Response:** All data has been included in ‘Supporting Information, S6’

d. **Provide other sources of articles**

If data or supporting information were obtained from another source (e.g. correspondence with the author of the original research article), please provide the source of data and dates on which the data/information were obtained by your research group.

**Response:** All research articles were obtained through Ulster University’s Open Access and library services.

e. **Provide the full assessment of Risk of Bias and Study Quality**

If applicable for your analysis, a table showing the completed risk of bias and quality/certainty assessments for each study or outcome. Please ensure this is provided for each domain or parameter assessed. For example, if you used the Cochrane risk-of-bias tool for randomized trials, provide answers to each of the signaling questions for each study. If you used GRADE to assess certainty of evidence, provide judgements about each of the quality of evidence factor. This should be provided for each outcome.

**Response:** The final outcomes of all quality and risk of bias assessments are available in Supporting Information, S7. A complete breakdown of each individual parameter within each of the articles is available in Appendix A1. The following sentence has been added to the manuscript “The summary of quality and risk of bias assessment scorings are available in Supporting Information, S7, with full information available in Supporting Information, S11”.

f. **Add a section to the manuscript on missing data**

An explanation of how missing data were handled.

**Response:** The following sentence has been added to the methods section: “Articles with missing data were excluded from relevant analysis if the necessary information was unavailable”.

3. **Amend Funding Statement**

Please state what role the funders took in the study.  If the funders had no role, please state: ""The funders had no role in study design, data collection and analysis, decision to publish, or preparation of the manuscript."" Please include this amended Role of Funder statement in your cover letter; we will change the online submission form on your behalf. Please remove any funding-related text from the manuscript and let us know how you would like to update your Funding Statement.

**Response:** The Role of Funder statement in the cover letter has been amended to include "The funders had no role in study design, data collection and analysis, decision to publish, or preparation of the manuscript". No funding related text is present in the manuscript.

**Reviewer 1 Corrections:**

1. **Check data for medication, smoking, ethnicity, disease duration, socio-demographic (education level, income)**

- No meta-analysis, cross-sectional data, and confounders left unexamined limit the strength of the inferences.
- The manuscript could be improved (meta-analysis, confounding variables, and causal interpretations) if the authors clarify the limitations.
- Outcome consistency is affected by heterogeneity among included studies (e.g., different methodologies, diagnostic criteria, and population characteristics). The reliance on cross-sectional studies (87 of 109 studies) precludes an established causal relationship.

**Confounders Response:** Added in the following paragraph to the limitations section in the discussion: “Finally, most of the included studies did not hold data for correlation between men and women, stratified smoking status, medication usage and socioeconomic status. While never smokers’ data was used for the regression analysis, only one paper provided definitive groupings according to the diabetes medication liraglutide [115], no article could be split into educational status in line with our participant groupings and only one article provided information on occupational status [55]. However, similarly, this information could not be categorised according to the groupings for this investigation. Six papers provided male and female data that could be separated according to this article’s groupings [36,40,62,64,76,92], but only two of these were Type 2 diabetes articles. Consequently, there was insufficient data to allow for analysis of these potential confounding variables”.

Also added the following sentence to the methods section “Being from Europe or North America and not having Type 2 diabetes were used as the reference standards, as ethnicity data was insufficient for regression analysis”.

**Meta-Analysis Response:** In the results section: “Due to the limited number of Type 2 diabetes studies containing a defined BMI range (n=1 lean; n=1 overweight; n=3 obese) it was not possible to separate Type 2 diabetes data based on BMI and correlate it directly with lung function data, nor was it possible to perform meta-analysis.”.

Also included in the limitation section in the discussion: “Furthermore, due to there only being one article that separated diabetes into lean, overweight and obese categories [78], it was not possible to perform a meta-analysis, heterogeneity analysis nor publication bias analysis.”.

**Cross-sectional Response:** Included in the limitations: “Most of the included studies were cross-sectional studies. The data for these studies were taken at only one time point and cannot account for any causal relationship. Thus, although many cross-sectional studies conclude there is accelerated lung dysfunction in diabetes, some longitudinal studies suggest there is no acceleration in decline after diabetes diagnosis [140].”.

**Heterogeneity Response:** Included in the limitations: “Heterogeneity in study methodologies can significantly impact the consistency of outcomes across included studies, as is seen here in the recording of FEV1/FVC measurements. Typically, restrictive and obstructive lung diseases are diagnosed by FEV1/FVC (L/L) ratio, and not by FEV1/FVC% of predicted. Unfortunately, not all studies stated which measurement they used; therefore, if the measurement used was not clear, it was assumed to be L/L.”

2. **Check data for obesity claims**

- Most conclusions are backed by the data, but more work is required to validate some claims (like that obesity plays a role in lung function).
- This publishing goes against the findings from several previous studies showing how obesity is harmful with regard to lung function decline. The authors recognize this but are not sufficiently clear about what might explain the difference.

**Response:** The following paragraph has been added to the discussion: “In this analysis, BMI had no significant impact on lung function in people without a preexisting lung condition. This is generally in contrast to existing literature [34,37,45,52,64,90,102]. The findings in this article may be due to the vastly different population ethnicities in the included studies. Additionally, central obesity may be a stronger indicator of lung function than BMI [53]. Despite this finding, weight-loss improves spirometry measurements in obese people both with and without asthma [129,130]. In the presence of a lung disease, BMI not having a significant impact on lung function may be explained by the fact that lung function has already been diminished as a result of the underlying lung disease. Significant impairment is already occurring and therefore limiting the capacity for further deterioration. Consequently, the underlying lung disease pathology may be eclipsing the impact that obesity, and indeed T2DM, may be having on the lungs. In the presence of Type 2 diabetes, as the data in this instance could not be analysed according to BMI, the linear regression analysis was performed to address this”.

3. **Clarify publication bias**

The trial does not descriptively assess for publication bias that may affect the reported findings.

**Response:** The following sentence has been added to the limitations: “Furthermore, due to there only being one article that separated diabetes into lean, overweight and obese categories [78], it was not possible to perform a meta-analysis, heterogeneity analysis nor publication bias analysis.”

**Reviewer 2 Corrections:**

1. **Check opening introduction sentence**

Review the first line of the introduction to ensure the wording “...Type 2 diabetes is due to microvascular and microvascular complications” is correct.

**Response:** Microvascular was incorrectly repeated, when it should have said macrovascular. The introduction sentence has been re-worded to “Microvascular and macrovascular complications significantly contribute to the morbidity and mortality associated with Type 2 diabetes”.

2. **Explanation why there is a difference in numbers from 109 to 170**

In the results section, subtitled “Association of Type 2 diabetes and/or obesity with lung function: Review of the literature”, it is mentioned “Initial analysis was performed on all studies...”. Please clarify the discrepancy between the total number of articles to be analysed and the breakdown of articles in this section.

**Response:** The total 93 articles had multiple conclusions. There is a larger number referenced in this section as several articles have been included more than once, due to them having more than one relevant conclusion to this review. The last referenced number in Table 1 is 114 and the first referenced number in the discussion section is 115. Therefore, the only articles referenced in the “Association of Type 2 diabetes and/or obesity with lung function: Review of the literature” section are the 93 articles already included in the Table 1 references.
